# Vitrimer-Like Shape Memory Polymers: Characterization and Applications in Reshaping and Manufacturing

**DOI:** 10.3390/polym12102330

**Published:** 2020-10-12

**Authors:** Tao Xi Wang, Hong Mei Chen, Abhijit Vijay Salvekar, Junyi Lim, Yahui Chen, Rui Xiao, Wei Min Huang

**Affiliations:** 1 College of Aerospace Engineering, Nanjing University of Aeronautics and Astronautics, 29 Yudao Street, Nanjing 210016, China; wa0003xi@nuaa.edu.cn; 2 College of Chemistry and Materials Science, Sichuan Normal University, Chengdu 610066, China; 3 School of Mechanical and Aerospace Engineering, Nanyang Technological University, 50 Nanyang Avenue, Singapore 639798, Singapore; ABHIJITV001@e.ntu.edu.sg (A.V.S.); li0020yi@e.ntu.edu.sg (J.L.); 4 School of Physical Science and Technology, Soochow University, Suzhou 215006, China; chenyahui@suda.edu.cn; 5 Key Laboratory of Soft Machines and Smart Devices of Zhejiang Province, Department of Engineering Mechanics, Zhejiang University, Hangzhou 310027, China; rxiao@zju.edu.cn

**Keywords:** vitrimer, shape memory, cross-linking, reversible, 3D printing, reshaping

## Abstract

The shape memory effect (SME) refers to the ability of a material to recover its original shape, but only in the presence of a right stimulus. Most polymers, either thermo-plastic or thermoset, can have the SME, although the actual shape memory performance varies according to the exact material and how the material is processed. Vitrimer, which is between thermoset and thermo-plastic, is featured by the reversible cross-linking. Vitrimer-like shape memory polymers (SMPs) combine the vitrimer-like behavior (associated with dissociative covalent adaptable networks) and SME, and can be utilized to achieve many novel functions that are difficult to be realized by conventional polymers. In the first part of this paper, a commercial polymer is used to demonstrate how to characterize the vitrimer-like behavior based on the heating-responsive SME. In the second part, a series of cases are presented to reveal the potential applications of vitrimer-like SMPs and their composites. It is concluded that the vitrimer-like feature not only enables many new ways in reshaping polymers, but also can bring forward new approaches in manufacturing, such as, rapid 3D printing in solid state on space/air/sea missions.

## 1. Introduction

**Figure 1:**
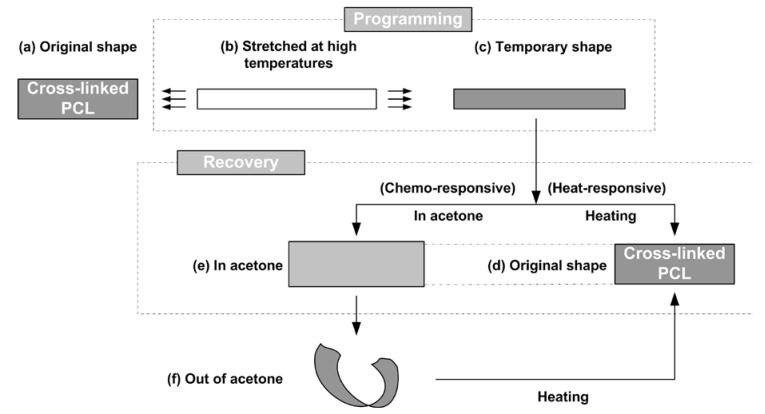
Shape memory effect in thermoset polycaprolactone (PCL). (**a**) Original strip shaped sample; (**b**) stretching at high temperatures when the material is soft; (**c**) temporary shape after programming; (**d**) recovered shape after heating; (**e**) recovered shape after immersing in acetone (slightly swollen); (**f**) after taking out of acetone, sample curls because of uneven evaporation of acetone. [(**b**)–(**c**): Programming to fix the temporary shape; (**c**)–(**d**): recovery via heat-responsive SME; (**c**)–(**e**): recovery via chemo-responsive SME; (**f**)–(**d**) heating to eliminate the deformation induced during acetone evaporation].

As a typical thermo-plastic, above its melting temperature (T_m_), polycaprolactone (PCL) is able to flow and can be reshaped into another shape. This new shape remains after solidification [1]. We can repeat this reshaping process again and again. However, after cross-linking, the PCL strip (refer to Figure 1) pre-stretched above its melting temperature is able to recover its original shape upon either heating or immersing in acetone. After pre-deformation to fix a temporary shape, a process called programming (e.g., Figure 1b to Figure 1c), a material with the ability to recover its original shape, but only at the presence of a right stimulus, is technically called shape memory material (SMM), and the phenomenon associated with this shape recovery process (e.g., Figure 1c to Figure 1d, which is heat-induced, or Figure 1c to Figure 1e, which is chemically induced) is known as the shape memory effect (SME) [2,3,4,5]. Similar to drying of wet hydrogel membranes in air [6], during drying of acetone wetted PCL, uneven evaporation of acetone may cause the PCL to curl (Figure 1e to Figure 1f) and an internal stress field is resulted inside of the material. Subsequent heating may effectively flatten it (Figure 1f to Figure 1d) and eliminate the internal stress as well. Refer to Figure S1 in the Supplementary Materials (Part I) for a snapshot of the actual experiment. After cross-linking, PCL becomes thermoset, and therefore its permanent shape is fixed [7,8,9]. It has both the heating-responsive SME and the chemo (acetone)-responsive SME. Furthermore, as revealed in S2 (thermo-plastic polystyrene, PS and thermo-plastic polypropylene, PP) and S3 (dry thermoset hydrogel) in the Supplementary Materials (Part I), the local internal stress in a thermoset can be eliminated or minimized via the SME, while for thermo-plastics, not only the local internal stress is difficult to remove, but also melting induced distortion may occur.

Although thermoset is well-known for better thermal/shape stability and higher strength, being reprocess-able via injection/extrusion etc., at high temperatures (above T_m_) is the remarkable advantage of thermo-plastic. Because of the increasing demand for recycling, being reprocess-able becomes more and more important at present [10,11]. 

Both the glass transition and melting/crystallization may be utilized for the SME in shape memory polymers (SMPs). For the melting/crystallization-based SME in a SMP, cross-linking, either physical or chemical, which determines the permanent shape of the SMP, is required [12,13]. There are different ways to describe the underlying mechanisms for the SME in SMPs. From the mechanics of materials point of view, there are at least two parts in a SMP, one is the elastic part, which stores the elastic energy after programming and releases the elastic energy to drive shape recovery, and the other is the transition part, which changes its stiffness according to whether the right stimulus is presented and hold the deformed temporary shape after programming. Depending on the actual working mechanism, the elastic part might be the cross-linked network only, or together with additional contribution from a portion of yet softened transition part during programming [14,15,16].

It is apparent that a reversible cross-linking system/network, which appears and disappears on demand in a well-controlled manner via, e.g., heating/cooling, is able to combine the advantages of both thermoset and thermo-plastic, and in the meantime, largely avoid their own problems. The potential application of this kind of material is enormous. 

Vitrimer is a newly coined term for this type of polymers with a reversible cross-linking system/network upon thermal cycling [17,18,19]. Although strictly speaking, according to [20], vitrimer belongs to the group of associative covalent adaptable networks (CANs) (i.e., the original cross-link is only broken when a new covalent bond to another position has been formed [20]), the group of dissociative CANs (i.e., the chemical bonds are first broken and then formed again at another place, for instance the reversible Diels–Alder reaction between furans and maleimides in organic polymer networks [20]) is seemingly more interesting and useful. Interested readers may refer to above mentioned references for more details. 

In recent years, many new types of vitrimers have been invented via different approaches for enhanced performance and/or for some special features (such as, the novel SME or self-folding/unfolding [21,22,23,24,25], i.e., reshaping), malleability and reprocessability by hot-pressing (i.e., associated with recycling as thermo-plastics) and heat-assisted healing, etc., [21,25,26,27,28,29,30,31,32,33,34,35]. Current methods to characterize vitrimers are mostly based on the Arrhenius law [17,33,35]. 

Some dissociative CAN vitrimers have been available in the market for some years. We used a commercial vitrimer polyurethane (PU) in the course of this study.

Upon thermal cycling within a relatively lower temperature range, if the network (cross-linking) in a vitrimer is strong enough to serve as the elastic part to store elastic energy, similar to those semi-crystalline SMPs, the heating-responsive SME can be achieved [12]. Since the cross-linking in vitrimers is reversible during thermal cycling to higher temperatures, we may examine the shape memory performance of a vitrimer to systematically characterize its vitrimer-like behavior (dissociative CAN) as a function of temperature. From the engineering application point of view, such a kind of information is essential in order to use the material in real practice.

The purpose of this paper is two-fold. Section 2 presents a heating-responsive SME based approach to systematically characterize the vitrimer-like behavior of a commercial vitrimer-like polymer. Section 3 presents a range of ways to permanently or temporarily reshape/manufacture vitrimers and their composites, including rapid 3D printing on space/air/sea missions. Main conclusions are summarized in Section 4.

Unless otherwise stated, in all experiments mentioned in Section 3, the vitrimer-like polymer characterized in Section 2 is used.

## 2. Characterization of Vitrimer-Like Behavior via SME

### 2.1. Material and Sample Preparation

The polymer (TPU 262A) used in the course of this study is from Taiwan PU Corporation (TPUCO, Taiwan). This material is claimed to be thermo-plastic by the manufacturer. As-received material is in pellet form. 

**Figure 2:**
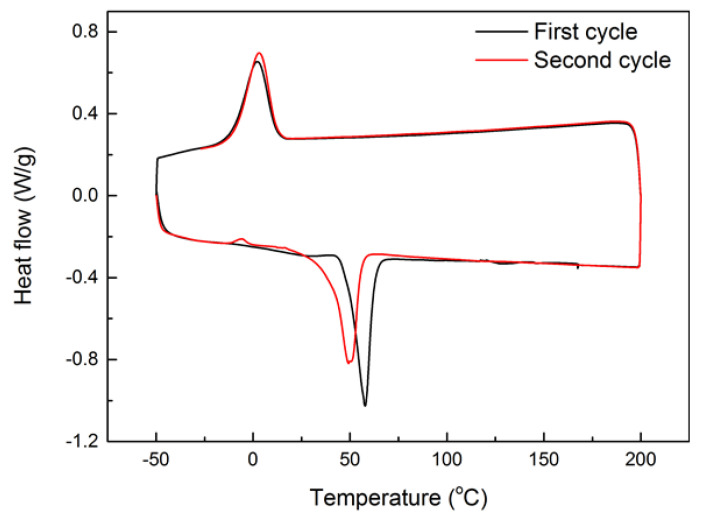
Differential scanning calorimetry (DSC) result of as-received pellet for two continuous thermal cycles.

Differential scanning calorimetry (DSC) test was carried out on a piece of small original pellet for two continuous thermal cycles between −50 °C and 200 °C at a temperature ramping rate of 10 °C/min using a Q200 DSC machine (TA Instrument, New Castle, DE, USA). According to the result of the second cycle (red line in Figure 2), the melting temperature (T_m_) of this material is about 50 °C, and at around 60 °C it fully melts. Upon cooling, its crystallization temperature (T_c_) is found to be about 0 °C. However, after 15 min at room temperature (about 23 °C), which according to Figure 2, is slightly above the crystallization starting temperature of this material, it is able to fully crystallize. An additional DSC test carried out between −100 °C and 200 °C (Figure S4 in the Supplementary Materials, Part I) reveals that the glass transition temperature range of this material is between −50 °C and −30 °C.

**Figure 3:**
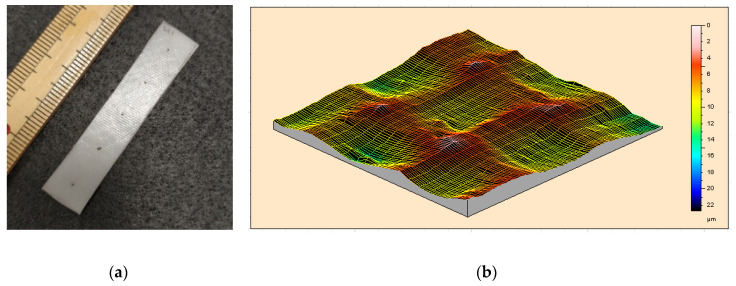
Typical strip-shaped 1-mm thick sample prepared by hot-compression (**a**) (standard rule is included for reference) and 3D surface morphology (**b**) (scanned area: 1 mm × 1 mm, the unit for the scale bar is μm).

Flat pieces with a thickness of 1 mm or 0.3 mm were prepared via hot-compressing at 130 °C. It was observed that at 110 °C, this polymer is already very easy to flow (refer to Figure 5). Hence, unless otherwise stated, the applied pressure was always very small in all hot-compression tests reported here. Samples were cut from the hot-compressed pieces into the required size for testing. Polytetrafluoroethylene (PTFE) thin film was used as the interfacial layer between the polymer and the metallic mold to ensure easy separation after hot-compression. Consequently, the textile pattern of the PTFE film was recorded on the surface of the hot-compressed samples as shown in Figure 3a, which is a strip-shaped sample cut from the hot-compressed piece. A few dots were marked on the sample surface for the purpose of manual measurement of the real deformation during thermo-mechanical testing. Figure 3b reveals the 3D surface over an area of 1 mm × 1 mm scanned by Talyscan 150 (Taylor Hobson, Warrenville, IL, USA), in which the fluctuation in height of the surface of the hot-compressed sample is around 20 μm. Samples were cut from the hot-compressed pieces into the required size for testing. 

Some pellets were dissolved in acetone (99% purity) at a concentration of 20 g/100 mL and then kept on stirring for 24 h. Thereafter, the solution was poured into a Petri dish without covering for 24 h for complete evaporation of acetone. Flat pieces of about 1 mm in thickness were obtained in this way. Herein, the samples cut from this kind of flat pieces are named as acetone-treated sample, while the samples prepared by hot-compression are named without acetone-treated sample.

### 2.2. Characterization

A series of experiments, including nuclear magnetic resonance (Figure S5), X-ray diffractometry (XRD) (Figure S6), and Fourier-transform infrared spectroscopy (FTIR) (Figures S7 and S8) were conducted to identify the chemical structure of this material. Refer to Part II of Supplementary Materials for details.

According to the ^1^H NMR spectrum in Figure S5 (Part II in the Supplementary Materials), this TPU is composed of poly(1,4-butylene adipate glycol) (PBA), diphenyl-methane-diisocyanate (MDI), and 1,4-butylene glycol (BDO). PBA segments form the soft domain (crystalline), while MDI and BDO segments form the hard segments (glassy). Refer to Figure S9 (Part II in the Supplementary Materials), the soft/hard segment structure enables the heating-responsive SME in this material at relatively lower temperatures (below 80 °C), in which the soft segments serve as the transition part, while the hard segments work as the elastic part.

Unless otherwise stated, herein the stress and strain are meant for the engineering stress and engineering strain, respectively. 

**Figure 4:**
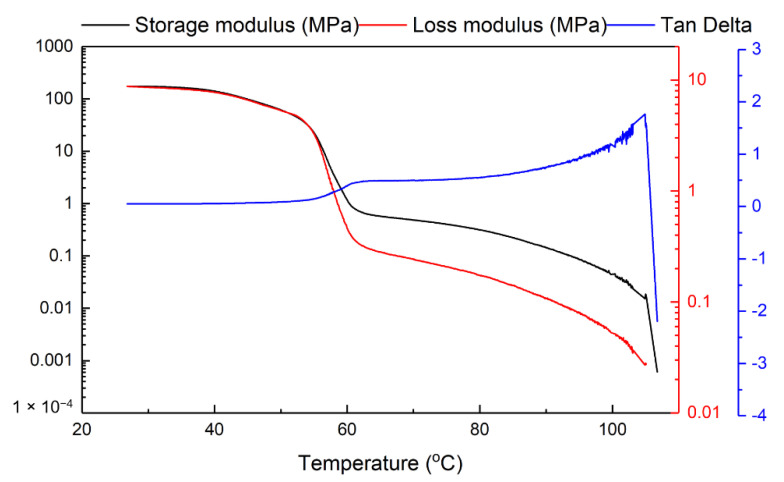
Typical dynamic mechanical analysis (DMA) result of sample without acetone treatment.

**Figure 5:**
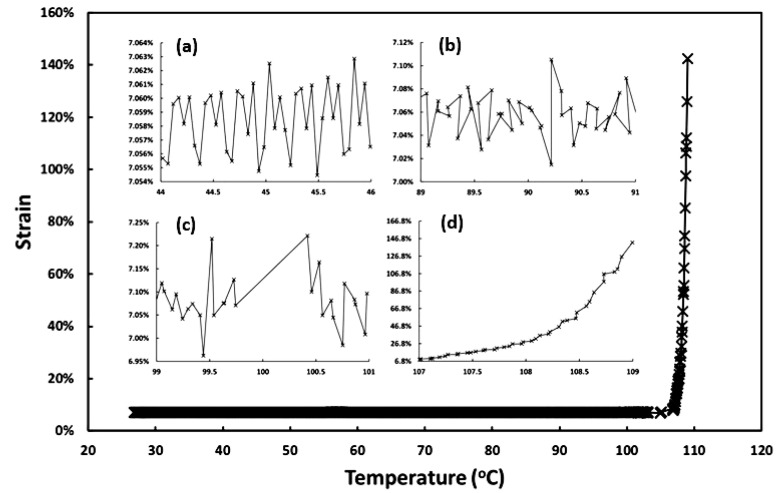
Strain versus heating temperature relationship in DMA test (same set of experimental data for Figure 4). (**a**): Zoom-in view from 44 °C to 46 °C; (**b**) zoom-in view from 89 °C to 91 °C; (**c**) zoom-in view from 99 °C to 101 °C; (**d**) zoom-in view from 107 °C to 109 °C.

A Q800 DMA machine from TA Instruments (New Castle, DE, USA) was used for all dynamic mechanical analysis (DMA) tests (in film tension mode at a ramping rate of 1 °C/min from room temperature to 115 °C). The applied frequency and oscillation strain were 1 Hz and 0.07%, respectively. The samples used here are 20 mm × 5 mm × 1 mm strips. The DMA result presented in Figure 4 is for the sample without acetone treatment, which confirms that the melting transition of this material ends at around 60 °C. In Figure 5, we plot the strain versus heating temperature relationship using the same set of experimental data for Figure 4. Four zoom-in views of four selected temperature ranges are also included. Although we cannot see any significant feature in both Figure 2 (DSC) and Figure 4 (DMA) at around 100 °C upon heating, Figure 5 [in particular, inset (d)] clearly shows that from 107 °C onward the material becomes more and more non-elastic and continuously extends during cyclic stretching. Hence, the material gradually turns to be less viscous, i.e., with less friction. At around 110 °C, the material appears not being able to maintain its shape anymore, so that the material becomes thermo-plastically reprocess-able.

**Figure 6:**
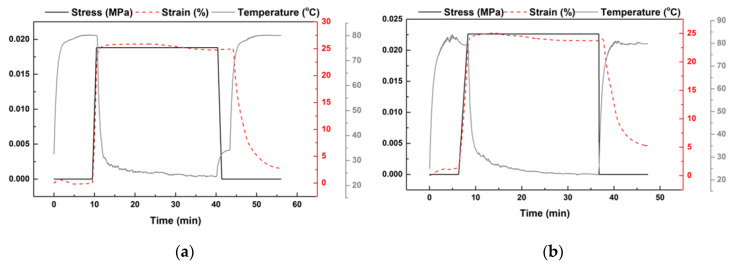
Typical DMA results for characterization of shape memory performance (T_d_: 80 °C; *ɛ_m_*: ~25%). (**a**) Acetone-treated sample; (**b**) without acetone treatment.

The heating-responsive shape memory performance of the samples with/without acetone treatment was characterized via DMA. In each test, the 1-mm thick sample was heated to a prescribed temperature. After three minutes of temperature stabilization, the sample was stretched to about 25% strain at a strain rate about 0.2%/s. After cooling back to room temperature, the sample was unloaded. This ends the programming process, which is the first part of a full shape memory cycle. Technically speaking, this heating temperature is called the programming temperature (T_d_) and the maximum deformation strain applied here is called the maximum programming strain (*ε_m_*) [36]. In the next part of a full shape memory cycle, which is the recovery process, the programmed sample was heated to the previous programming temperature (T_d_) for shape recovery. Two typical DMA results, in which T_d_ was 80 °C in both tests, but one sample was acetone treated and the other was without acetone treatment, are presented in Figure 6. Since the horizontal axis is time, the stress, strain, and temperature during each shape memory cycle are presented as a function of time.

Two most important parameters to evaluate the shape memory performance of a polymer are the shape fixity ratio (*R_f_*) and shape recovery ratio (*R_r_*), which, for polymers without apparent creep/relaxation at room temperature after programming (as the material used in this study is; refer to [37] for the experimental results of this material in our previous investigation) are defined by [36],

(1) $$R_{f}=\frac{\varepsilon_{1}}{\varepsilon_{m}}$$

(2) $$R_{r}=\frac{\varepsilon_{1}-\varepsilon_{2}}{\varepsilon_{1}}$$

where *ɛ*_1_ is the strain after programming and *ɛ*_2_ is the residual strain after heating for shape recovery. As mentioned in [12], both the shape fixity ratio and shape recovery ratio of a polymer are programming temperature and maximum programming strain dependent.

**Figure 7:**
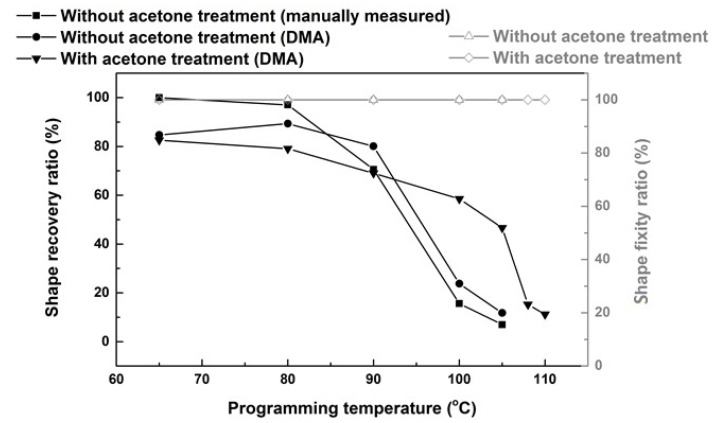
Shape fixity ratio (*R_f_*) and shape recovery ratio (*R_r_*) as a function of programming temperature (T_d_) for samples with/without acetone treatment.

In Figure 7, *R_f_* and *R_r_* (upon heating to T_d_, which is the programming temperature, for shape recovery) are plotted as a function of T_d_. As the material around the clamping areas might be pulled out of the clampers during stretching, which is difficult to recover in the subsequent heating process in a DMA test, the shape recovery ratios of the samples without acetone treatment obtained based on the displacement manually measured using the pre-marked dots in the middle area of these samples (refer to Figure 3a) are also included in Figure 7. It appears that *R_f_* in all samples is about 100%, while *R_r_* decreases with the increase in T_d_. Both acetone-treated and without acetone-treated samples essentially share the same trend in the *R_r_* versus T_d_ relationship_._ The manually measured results of without acetone-treated samples indicate that for T_d_ up to 80 °C, upon heating to the previous T_d_, the corresponding *R_r_* is about 100%. When T_d_ approaches 110 °C, the corresponding *R_r_* drops to only about 10% or less in all experiments. The results of *R_r_* obtained from DMA test and manual measurement are only slightly different. 

Additional tests were manually carried out to further investigate the influence of thermal history on *R_r_* using 1-mm thick samples. Manual measurement based on pre-marked dots (refer to Figure 3a) was used in the calculation. All these tests were carried out according to the following sequence.

**Figure 8:**
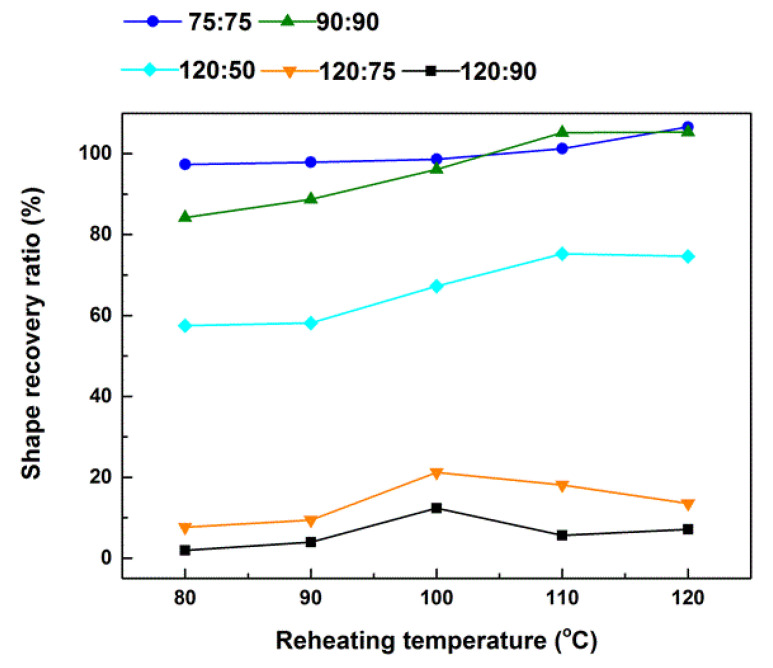
Relationship between shape recovery ratio (*R_r_*) and reheating temperature (T_r_) for pre-heated (to T_p_) and then deformed (upon cooling to T_d_) samples. Legend is in (T_p_:T_d_) format, where T_p_ and T_d_ indicate the pre-heating and programming temperatures, respectively, of a sample.

(1) Heat the sample to a prescribed pre-heating temperature (T_p_). Three pre-heating temperatures (namely, 75 °C, 90 °C, and 120 °C) were applied in the course of this study.
(2) Cool the sample to a predetermined programming temperature, T_d_ (≤T_p_), and then gently and slowly stretch it by hands so that the strain between the two marked dots reaches around 100%. Three programming temperatures (namely, 50 °C, 75 °C, and 90 °C) were applied. According to Figure 2, these three temperatures are all well above the crystallization temperature of this material.
(3) Further cool the sample back to room temperature for full crystallization.
(4) Heat the sample in a step-by-step manner to five reheating temperatures (T_r_) from 80 °C, 90 °C, 100 °C, 110 °C, to finally 120 °C. The corresponding shape recovery ratios after each heating are plotted in Figure 8. Legend in Figure 8 is in (T_p_:T_d_) format to indicate the actual thermal history before final step-by-step heating of a sample.

Hence, Sample (75:75) was pre-heated to 75 °C and then programmed at 75 °C. Same as that in Figure 7, it is able to mostly recover its original shape upon heating to 80 °C. However, upon further heating to 110 °C and above, *R_r_* is slightly over 100%, which should be due to the easy to flow nature of this material at such high temperatures (refer to Figure 5d). For Sample (90:90), its *R_r_* is about 82% upon heating to 80 °C. Further heating to its programming temperature of 90 °C, *R_r_* improves slightly. With further increase in heating temperature, *R_r_* gradually and continuously increases until around 110 °C, where the material becomes dimensionally unstable. For the other three samples, they were pre-heated to 120 °C and then programmed at lower temperatures. Apparently, Sample (120:90) has very much limited capability for shape recovery (less than 10%), while Sample (120:75) is slightly better (20% or less). The shape recovery ratio of Sample (120:50) is over 50%, which is much better than Sample (120:75) and Sample (120:90). According to [12], a higher shape recovery ratio implies more/better elastic part, which releases the elastic energy stored during programming to drive shape recovery.

### 2.3. Vitrimer-Like Behavior

According to [12], the underlying mechanism for the SME in polymeric materials is a two-part/component system, in which one (elastic part) is always elastic to store elastic energy after programming and then the stored elastic energy serves as the driving force for later on shape recovery, while the other (transition part) is able to change its stiffness to function as the switcher in a shape memory cycle. 

According to Figure S4 (Part II of the Supplementary Materials), the soft segment is PBA, and the hard segment is MDI-BDO. Figure S9 in Part II in the Supplementary Materials schematically illustrates the phase transition process upon heating. While below 80 °C, the SME of this material is the result of the soft/hard segment system, upon further heating to over 80 °C, the glassy hard segments gradually become viscous. Correspondingly, the network gradually disappears. Consequently, the shape recovery ratio of the polymer gradually decreases.

After combining the results in Figure 7 and Figure 8 together, we may conclude that below 80 °C, the material is similar to a semi-crystalline polymer, i.e., thermoset. The chemically or physically crosslinked network is essentially the elastic part. However, upon further heating the network starts to gradually weaken, which results in continuous decrease in the shape recovery ratio. Above 110 °C, the material becomes easy to flow, which indicates that the network is either weakened to the level that is not sufficient to keep the shape or fully removed (refer to the DMA result in Figure 5d). The reason for less recovery upon heating to T_d_ and then more recovery upon further heating [e.g., Sample (90:90)] is because the network is weakened (evidenced by higher strain and more fluctuation in Figure 5b,c). Therefore, further softening at higher temperatures (refer to DMA result in Figure 4) is required for more recovery. 

After heating to eliminate all the network, in the subsequent cooling process, the network starts to form gradually, but not so significant even upon cooling to 75 °C, as the corresponding shape recovery ratio is still less than 20%. Upon further cooling to 50 °C, the shape recovery ratio is dramatically increased to over 50%. 

**Figure 9:**
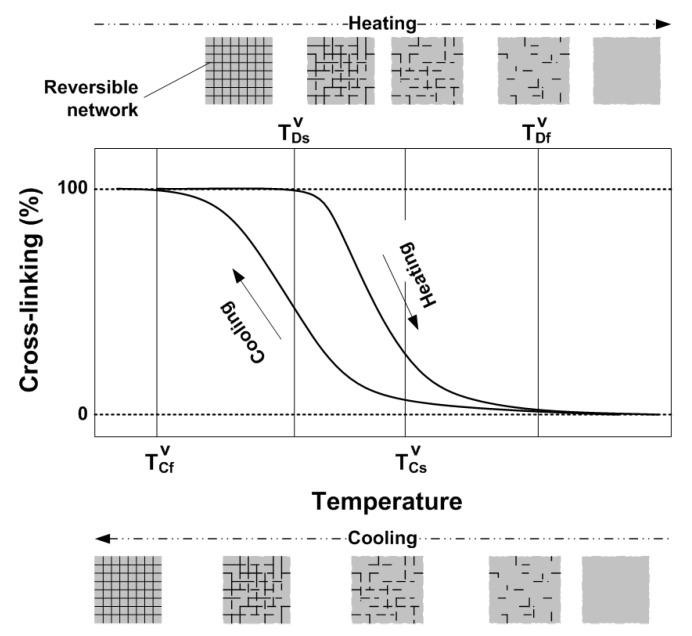
Schematic sketch of cross-linking (in %) versus temperature relationship upon heating (network eliminating) and cooling (gradual cross-linking).

It can be concluded that the reversible cross-linking in this material is dissociative CAN. In Figure 9, we schematically plot the relationship between cross-linking (in %) versus temperature in a thermal cycle. For the TPU investigated in this study, according to Figure S9 (Part II in the Supplementary Materials), the reversible network (cross-linking) is the hard segments (MDI-BDD) (Figure S5 in Part II of the Supplementary Materials), which gradually disappear upon gradual heating from 80 °C to about 110 °C. In the subsequent cooling process, the network slowly reappears.

In the heating process, the network starts to disappear upon reaching around $T_{Ds}^{V}$, which means that below this temperature, the material is 100% cross-linked and we may consider it as a kind of semicrystalline thermoset. Therefore, it can have excellent SME, i.e., being able to almost 100% recover its original shape, if the applied programming temperature and strain are right [12]. With the increase in temperature, the network gradually disappears, and hence the corresponding shape recovery is reduced accordingly. At about $T_{Df}^{V}$, the network is fully eliminated and consequently, the material becomes thermo-plastic and is easy to flow (i.e., with high melting flow index [37]). In the cooling process, cross-linking starts at around $T_{Cs}^{V}$ and at about $T_{Cf}^{V}$, the network reaches 100%. 

Based on Figure 9, it is expected that part of the network can be removed upon heating to between $T_{Ds}^{V}$ and $T_{Df}^{V}$. After subsequent cooling to $T_{Cf}^{V}$, the new cross-linking (100%) includes two parts, one is the remaining of the previous network, and the other is newly formed. By selecting the actual heating temperature between $T_{Ds}^{V}$ and $T_{Df}^{V}$, the fraction of the newly formed network can be tailored. If we heat the material to a temperature between $T_{Ds}^{V}$ and $T_{Df}^{V}$, and then stretch it (programming), after cooling, elastic energy is stored in the remaining of the previous network, while the newly formed network is mostly stress free.

The slight difference observed in Figure 7 between samples with/without acetone treatment implies the density of the reversible network is roughly the same before and after treatment (thermal cycling or dissolving in acetone).

In the heating process, dramatic decrease in shape recovery is observed, when the remaining network is not able to serve as a strong continuous elastic spring, which should follow the percolation theory, to effectively store elastic energy. 

**Figure 10:**
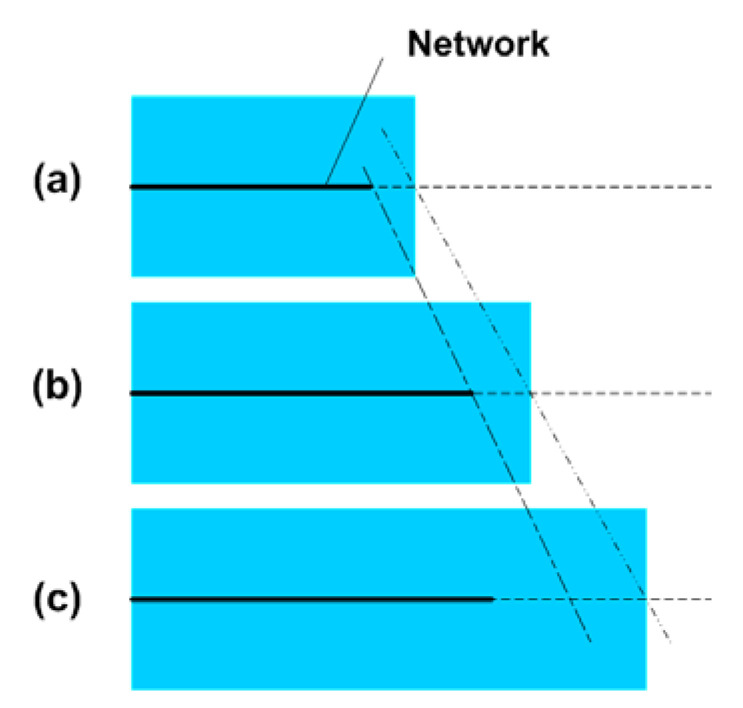
Schematic illustration of dislocation at the end of a network due to over-stretching. (**a**) Original (without stretching); (**b**) stretched within elastic limit; (**c**) over-stretched, dislocation which is permanent, occurs at the end of the network (the network is less extended).

Apparently, the programming strain also affects the continuity of the elastic spring (network). For instance, over stretching may cause one functionally continuous elastic spring to split into two or more smaller springs, which results in permanent plastic deformation and hence the reduced shape recovery capability of the material. As schematically illustrated in Figure 10a, if a material is slightly stretched at high temperatures, the network is able to elastically deform together with the matrix (Figure 10b). However, if it is over-stretched, dislocation, another type of permanent plastic deformation, at the end of the network occurs (Figure 10c), which reduces the shape recovery ratio accordingly. In the cooling process, the percolation theory for the relationship between the network and shape recovery capability is also applicable. Such a kind of relationship in both heating and cooling processes provides the possibility to simultaneously reset both the permanent shape and temporary shape of a vitrimer in one processing step.

For simplicity, in above mentioned experimental investigation, we only take the heating/cooling temperature as the parameter without considering the influence of heating/cooling time/speed. Since this material is able to fully crystallize at room temperature, which is slightly above its crystallization starting temperature (Figure 2), it is logical to expect that heating/cooling time/speed should be important factors as well.

It is clear that this polymer is indeed a vitrimer-like polymer (dissociative CAN), in which there is a network that can be gradually eliminated upon heating to over 80 °C that is above its melting finish temperature (according to Figure 2 and Figure 4), and upon cooling, the network gradually reappears. The shape recovery ratio (*R_r_*) essentially reveals the evolution of the network structure of a polymer, and may be utilized to systematically characterize the vitrimer-like behavior of a polymer upon thermal cycling. Such a kind of information is essential in order to apply the material in real engineering applications. It can be concluded that the *R_r_* versus programming temperature relationship of a vitrimer-like SMP (e.g., Figure 7) is programming strain and heating/cooling time/speed dependent. The relationship between R_r_ and cross-linking (in %) is nonlinear and sophisticated.

## 3. Applications of Vitrimer-Like SMPs and Their Composites

A combination of the vitrimer-like behavior and SME enables many new ways to manipulate polymers, far beyond folding/unfolding, reprocessing and heating assisted healing, which have been focused on in most of the studies reported so far [21,25,26]. In this section, in addition to these well-known applications, we explore some more possibilities in reshaping (inside/outside, microscopically/macroscopically, permanent/temporary) and manufacturing using vitrimer-like SMPs and their composites. Above characterized commercial vitrimer-like SMP is used to demonstrate all applications reported in Section 3.1, Section 3.2, Section 3.3, Section 3.4.

### 3.1. Superimposing of Permanent and Temporary Shapes

Although some ductile polymers may be programmed at low temperatures, for the heating-responsive SME, in most cases, it is ideal to carry out programming at high temperatures when the polymer is soft and ductile [14]. The transition that might be applied to soften a polymer includes the glass transition and melting transition. While the glass transition temperature ranges in the heating process and cooling process of a polymer are about the same for most polymers, the melting transition temperature range is normally well above the crystallization temperature range (refer to Figure 2 for an example). Programming a polymer based on the melting transition can be done either in the melting temperature range (or above) upon heating or in the crystallization temperature range (or above) upon cooling (after pre-heating). Hence, in the case of, for instance, comfort fitting in direct contact with human body [38,39], we are able to programme the polymer investigated in Section 2 at body temperature or even room temperature without worrying of being either too hot or short of time in programming (fitting), which are problems in those polymers in which the glass transition is utilized in programming.

**Figure 11:**
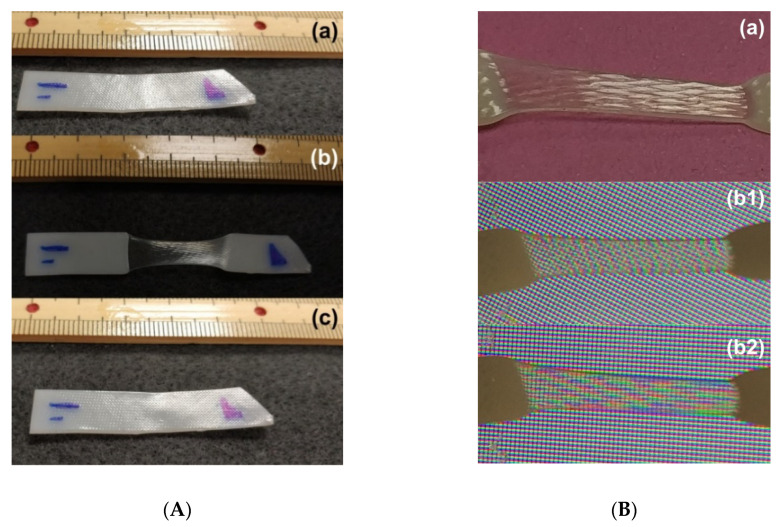
(**A**) Evolution of surface morphology in hot-compressed sample (1-mm thick) used in this study (refer to Section 2) in one shape memory cycle. (**a**) Original (opaque); (**b**) after stretching the middle part (transparent) of the preheated sample at room temperature; (**c**) after heating for shape recovery and cooling for crystallization at room temperature (opaque). (**B**) Coloring effect (light interference) in the middle-stretched area (transparent). (**a**) After stretching at room temperature; (**b1**,**b2**): sample with two different angles before LED computer monitor.

In Figure 11A, a piece of hot-compressed sample (without acetone treatment material) is pre-heated to its melting temperature (about 70 °C) and then hand-stretched when it is cooled to room temperature (about 23 °C) for a while. Since the middle part of the sample is stretched at lower temperatures, similar to the stress induced crystallization in PCL [1], i.e., the crystalline component increases remarkably after stretching, which results in improved transparency on account of minimized density differences between crystalline and amorphous regions, and hence reduced refractive index fluctuations [40], this area becomes transparent. After heating to the melting temperature again, it fully recovers its original shape (including the original surface pattern). Figure 11B reveals the typical surface morphology after stretching and the resulted coloring effect (light interference) due to the surface pattern in the middle transparent part, when it is placed in front of a LED computer monitor.

To reconfigure a polymer into another permanent shape without complete removal of the originally defined permanent shape is apparently an advantage of vitrimer [22,41,42]. According to Figure 9, reshaping into another permanent shape can actually be done during either heating or cooling when the network is partially or fully eliminated. Stretching under different conditions (depending on the thermal history and the way of deformation) may result in different colors (opaque or transparent) within the deformed area.

**Figure 12:**
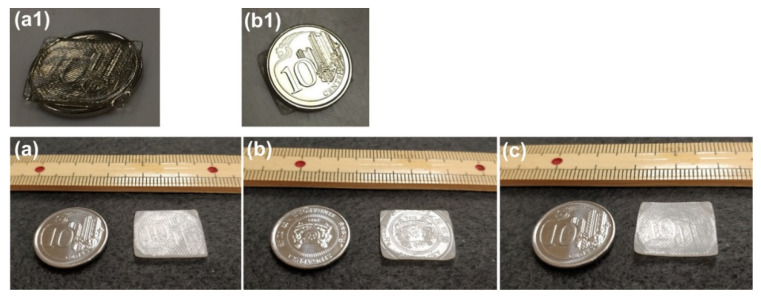
Superimposing new feature atop existing surface pattern. (**a**) After heating the sample (1 mm thick) placed atop a coin to around 100 °C [as shown in (**a1**), sample is fully transparent at high temperatures] to superimpose the surface pattern of the coin; (**b**) after pre-heated sample (to less than 80 °C) cooled to room temperature and then placing the other side of the coin atop for second impression [as shown in (**b1**)]; (**c**) after heating for shape recovery.

An example to superimpose a new permanent surface pattern (using a coin) atop the existing one (produced during sample preparation via hot-compressing as shown in Figure 3) is demonstrated in Figure 12, in which the first impression is done upon heating to around 100 °C (Figure 12(a1)), and the second impression is done after the pre-heated sample (to less than 80 °C) is just cooled back to room temperature (Figure 12(b1)). While the first impression (the mirror image of one side of a coin) is superimposed on the original permanent surface pattern produced during hot-compression (Figure 12a), the second impression (the mirror image of the other side of the coin) is temporary (Figure 12b), and can be fully removed after heating again (Figure 12c). Note that the maximum depth of the surface feature of a standard bank coin is about 0.1 mm [43,44], which is a lot more than that of the surface pattern of the hot-compressed sample (about 20 μm, refer to Figure 3b).

In Figure 12, in order to record the results after each step, the sample was heated twice to superimpose two surface patterns, one is permanent and the other is temporary, one by one. In fact, both the permanent pattern and temporary pattern can be superimposed within one heating-cooling process, i.e., the second surface pattern can be impressed onto the sample when the network is fully established in the cooling process. Furthermore, according to Figure 8 and Figure 9, we can actually superimpose a few permanent and temporary shapes during one heating-cooling process.

In solid state, vitrimer-like SMP has the advantage to reconfigure into another shape either permanently or temporarily on demand. 

### 3.2. Heat-Assisted Healing without Altering Surface Feature

Healing, in particular heat-assisted healing, has been extensively investigated in recent years (e.g., in [45,46,47,48,49,50]). In most applications, both shape recovery and strength recovery are simultaneously required. Ideally, healing should be repeatable and the healing process should take as less time as possible. 

For a thermo-plastic, unless heating is restricted within a very small local area, heating to or over its melting temperature tends to fail the material to maintain the original shape/dimension, in particular, surface pattern (refer to Figure S2(a2) in Part I of the Supplementary Materials). On the other hand, the broken network in a thermoset is mostly permanent. However, vitrimer (in particular for dissociative CAN type) is able to keep the original shape and reconstruct a network via heat-assisted healing for strength recovery in a repeatable and rapid manner. 

**Figure 13:**
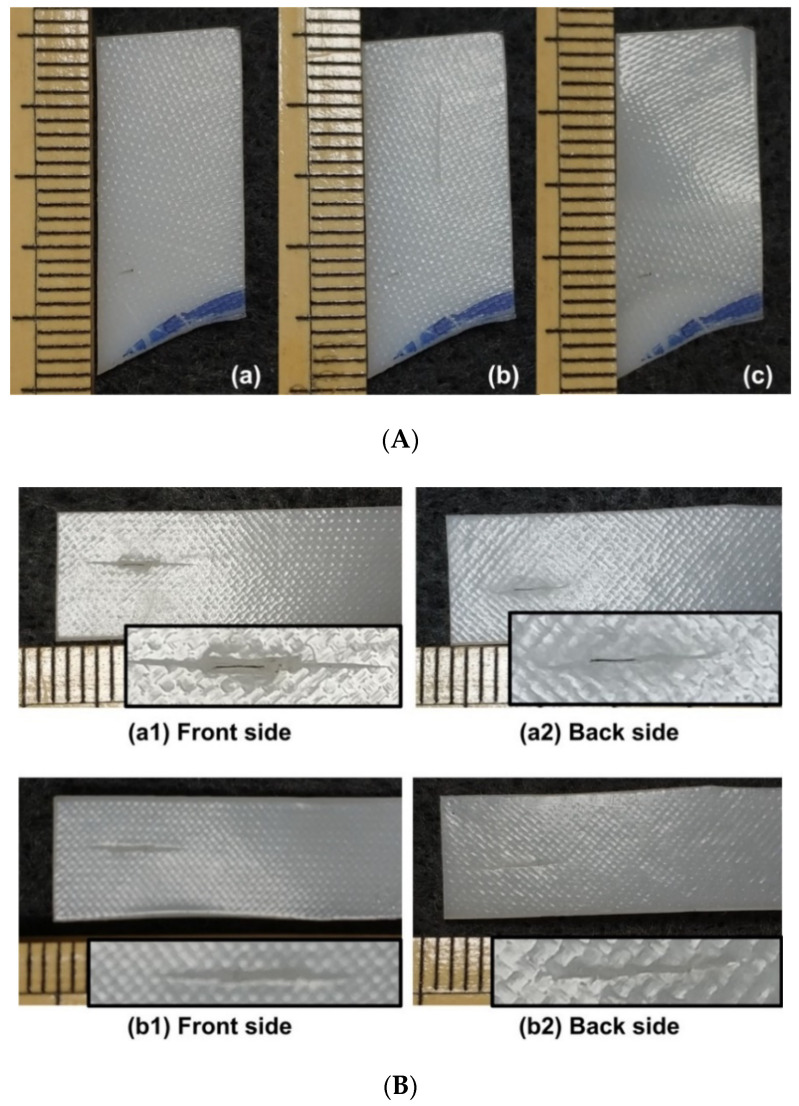
Heat-assisted healing. (**A**) Shallow surface cutting using sharp blade. (**a**) Original; (**b**) after cutting; (**c**) after heating in 90 °C hot water. (**B**) Throughout thickness cutting using pen-knife. Insets are zoom-in view of the cut. (a) After cutting; (b) after heating using hairdryer. (1) Front side; (2) back side.

Based on the characterized properties of the vitrimer-like SMP reported in Section 2, we can select an optimized temperature to heat the whole piece of a sample for healing within a short period of time, while the sample keeps the original shape/dimension. Two 1-mm thick examples as reported in Section 2 are demonstrated in Figure 13, in which Figure 13A is healing of surface cutting produced by a sharp blade via immersing into 90 °C hot water, and Figure 13B is healing of throughout thickness cutting produced by a pen-knife via heating using a hairdryer. According to Figure 2 and Figure 4, heating to 80 °C triggers the heating-responsive SME, which helps to close the cutting (shape recovery). Upon further heating to below its easy to flow temperature and then cooling back enables the network to be re-established, while the original surface pattern largely remains. The reversible network in vitrimer-like SMPs provides a temperature winder (between about 90 °C to about 100 °C for the material investigated in Section 2) to simultaneously achieve shape recovery and strength recovery with minimum impact on the original shape/dimension of the sample.

**Figure 14:**
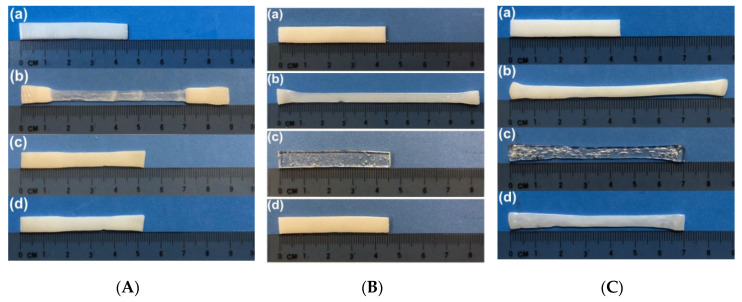
Typical SME tests programmed at three different temperatures. (**A**) Room temperature programming. (**a**) Original shape (opaque); (**b**) after stretching at room temperature (the middle part is transparent); (**c**) after heating to 80 °C (opaque); (**d**) after heating to 100 °C (opaque). (**B**) Programmed at 65 °C. (**a**) Original shape (opaque); (**b**) after programming at 65 °C (opaque); (**c**) upon heating to 65 °C for shape recovery (transparent); (**d**) after fully crystallized at room temperature (opaque). (**C**) Programmed at 95 °C. (**a**) Original shape; (**b**) after programming at 95 °C (opaque); (**c**) upon heating to 95 °C for shape recovery (transparent); (**d**) after cooling back to room temperature for crystallization (opaque).

In Figure 13, it appears that shape recovery is not 100%, in particular in the sample with throughout cutting. In order to reveal the exact reason behind this, further experiments were carried out on 1-mm thick samples. Figure 14 presents the results of three SME tests, in which the programming temperatures are room temperature, 65 °C and 95 °C, respectively, while the applied maximum programming strain is approximately the same. Since at room temperature the material is hard, stretching is done by a tensile machine. Programming of other two samples at higher temperatures is conducted by hands (in a similar way to investigate the influence of thermal history on *R_r_* in Section 2.2). As we can see, full shape recovery is only observed in the sample stretched at 65 °C, while the other two samples stretched at room temperature and 95 °C are not able to fully recover. As explained above, incomplete recovery in the sample programmed at 95 °C is due to the reason of significant reduction in cross-linking. On the other hand, for the sample programmed at room temperature, incomplete shape recovery after heating should be the result of fractured cross-linking when the sample is stretched at low temperatures, which is a phenomenon that has been reported [12,51]. Thus, it may be concluded that incomplete healing (shape recovery) observed in Figure 13 is most likely associated with the reduced shape recovery capability of the polymer after low temperature programming, since cutting is done at room temperature.

### 3.3. From 2D-Shape to Surface Wrinkling/3D-Shape

**Figure 15:**
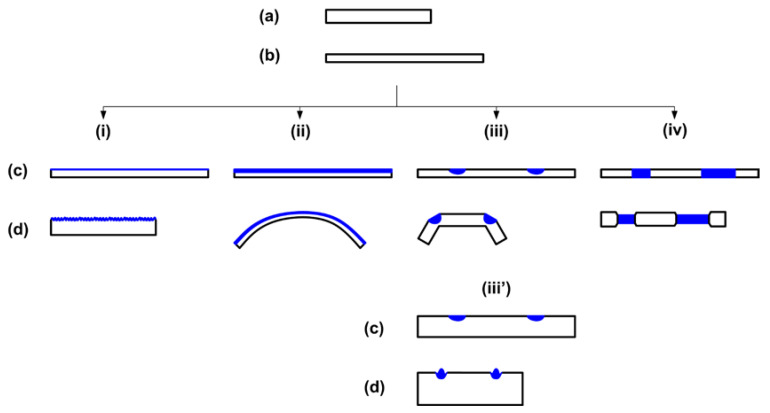
Illustration of typical approaches applicable for reshaping of vitrimer-like SMP strip (side view for all sketches) via gradient preheating and then heating for shape recovery. (**a**) Original shape; (**b**) after stretching; (**c**) after gradient heating; (**d**) after heating for shape recovery. (i) Wrinkling; (ii) bending; (iii) folding (local non-through thickness heating); (iii′) surface patterning (local surface heating); (iv) transition from uniform thickness to non-uniform thickness (local through thickness heating).

According to Figure 7 and Figure 8, the reversible network in this vitrimer-like SMP is gradually removed upon heating to above 80 °C till about 110 °C, where the material starts to flow (Figure 5). On the other hand, in the subsequent cooling process, the network gradually reinstalls. This feature can be applied via gradient heating (i.e., heating only selected area) to achieve surface wrinkling/patterning, 2D-shape to 3D-shape switching, and transition from uniform shape to non-uniform shape, etc., as schematically illustrated in Figure 15 using a piece of pre-stretched vitrimer-like SMP strip.

As presented in Figure 15, in general, there are three steps in all approaches. 

First (pre-deformation), the original strip (a) is programmed via stretching (b) preferably at high temperatures for better shape fixity ratio and higher shape recovery ratio (refer to Figure 14). Although illustrated here is the case of uniaxial stretching, programming may be carried out in other modes, such as biaxial stretching, compression, and twisting, etc., or a combination of them.

In the next step (c) (preheating), local/gradient heating is applied to eliminate the network in one (Figure 15i,ii) or more (Figure 15iii,iii′,iv) prescribed areas. After cooling back to room temperature, new network is formed without any elastic energy stored inside of it. In Figure 15(ci), the surface of the sample is preheated, while in Figure 15(cii), the preheated area is much deeper. 

Subsequently, upon heating for shape recovery, which is the third step (shape recovery), surface wrinkles are observed in Figure 15(di), while in Figure 15(dii), the sample bends toward the un-preheated side. 

**Figure 16:**
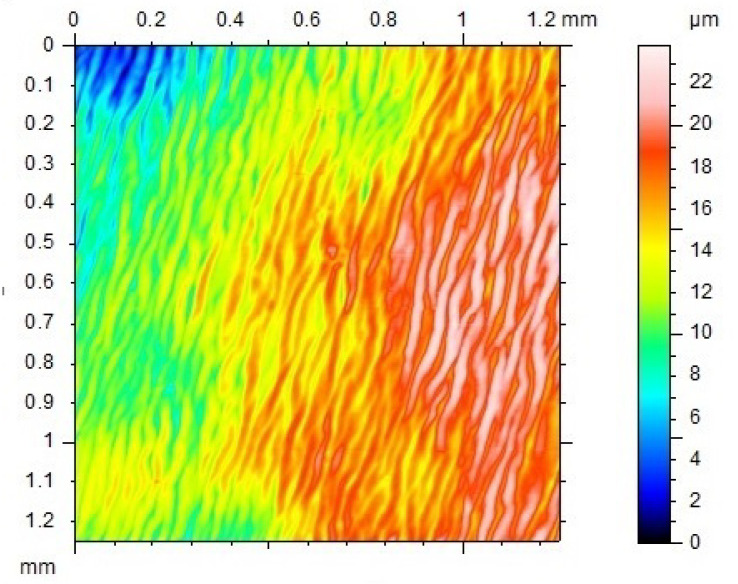
Surface wrinkles produced following approach (i) in Figure 15. The actual surface feature is a combination of initial surface pattern (refer to Figure 3) and wrinkles. In the preparation of the sample, a cotton swab was soaked in acetone and then applied to slightly etch the surface of 50% pre-stretched 1-mm thick hot compressed sample. Subsequently, the sample was heated in 75 °C water for shape recovery.

Figure 16 presents typical wrinkles produced on a 1-mm thick sample following approach (i) in Figure 15. The resulted parallel wrinkles look similar to what is reported in [52], in which pre-stretched acrylonitrile butadiene styrene (ABS) that is thermo-plastic was dipped in acetone to slightly etch its surface. After surface drying and then heating for shape recovery, parallel wrinkles were formed on the surface of ABS sample. In Figure S10 (Part III of Supplementary Materials), we schematically compare the difference between surface etching approach and surface preheating (vitrimer) approach. While surface preheating (vitrimer) approach is able to maintain the original surface pattern (as in Figure 16), surface etching approach can hardly keep the original surface structure, in particular if the surface feature is small in size. 

**Figure 17:**
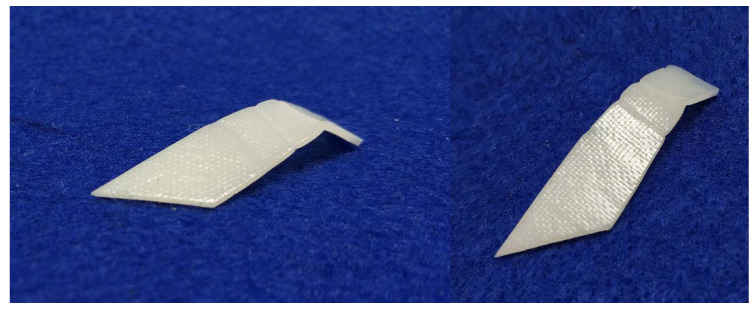
Folding following approach (iii) in Figure 15 (two different angles of view).

In Figure 15(ciii), instead of the whole surface layer, some local areas are preheated. Upon heating for shape recovery, the sample folds (Figure 15(diii)). Figure 17 is an example of such 2D to 3D folding after heating. The 1 mm thick sample was pre-stretched by 30%. Local heating (two straight lines) was done using a 3D printing pen (from eSUN, PR China) to slowly tough the sample surface during writing (without filament). The pen head was adjusted to be about 100 °C. After heating for shape recovery, the initial surface pattern produced during hot-compressing is still clearly visible. If the depth of the preheated area is relatively small, as illustrated in Figure 15(ciii′), the result might be a patterned surface as shown in Figure 15(diii′). Of course, preheating might be throughout the whole thickness of the sample (Figure 15(civ)), so that after overall-heating of the whole sample for shape recovery, some parts of the sample shrinks, while some other parts fully or partially maintain the shape (Figure 15(div)). It should be pointed out that it might be necessary to restrain the sample in step (c) of Figure 15 from undesired distortion during the last heating process.

**Figure 18:**
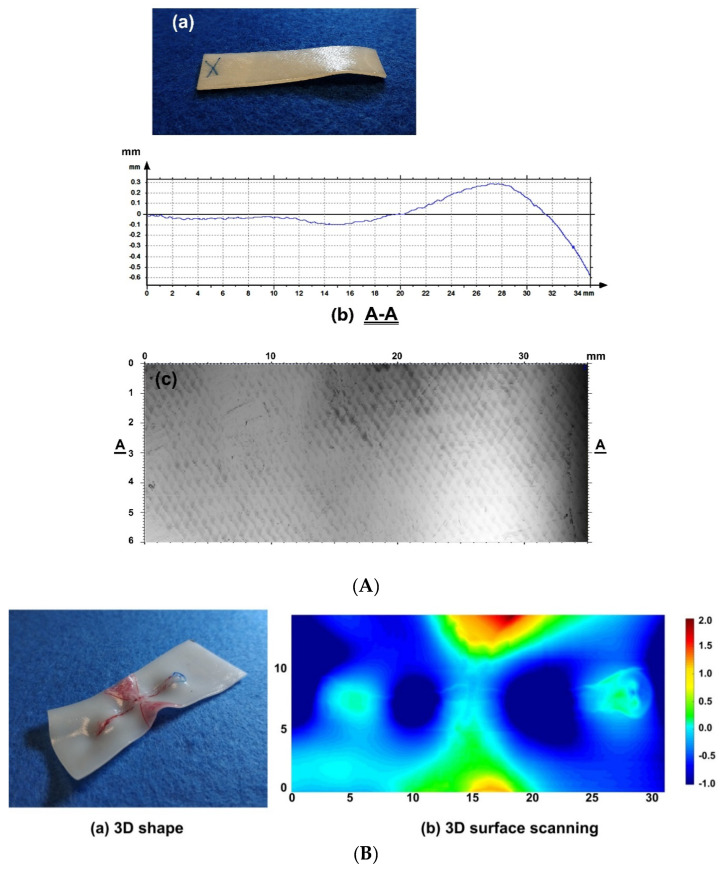
Controlled folding. (**A**) Partial-bending. (**a**) Photo; (**b**) 3D line scanning result (top surface) of A-A section; (**c**) top view, in which A-A section is marked. (**B**) 3D folding. (**a**) Photo of resulted shape (red mark pen colored areas are meant to guide for local heating); (**b**) 3D scanning result (unit is in mm for all).

A combination of the preheating methods mentioned above can result in different ways of folding. Figure 18 presents two examples of 2D to 3D folding. In Figure 18A, after pre-stretching to 20%, the right part of the top sample surface of a 1 mm thick sample is preheated to high temperatures using the same 3D printing pen mentioned in above. Thus, after 2nd heating for shape recovery, the right part of the sample bends down (Figure 18(Aa)). Figure 18(Ab) is 3D scanned cross-section of the line marked as A-A in Figure 18(Ac) (optical image, top view). We can see the original surface pattern largely remains. Figure 18B is a combination of surface preheating and deep-line preheating (again using the 3D printing pen) of a piece of pre-stretched 1-mm thick sample. The resulted 3D shape after heating for shape recovery can be pre-designed.

If a vitrimer is transparent, sophisticated patterns, e.g., integrated with wrinkles and/or other particular surface patterns, may be used as optical lens for anti-counterfeit applications [44]. Local heating to selectively release internal elastic stress in pre-deformed vitrimer to achieve so called digital coding has been reported in [53].

For simplicity, a strip is used for illustration in Figure 15. There are many other possible shapes for different applications.

Since upon heating to above its melting temperature but less than 80 °C, this vitrimer-like SMP is transparent and has excellent SME (Figure 14), local preheating may be carried out by laser engraving method in a 3D pixel manner, when the material is transparent.

### 3.4. Vitrimer Composites

While most previous works on vitrimer composites are focused on their capability of recycling and self-healing [54,55,56,57], rapid reshaping to fix a new permanent/temporary shape (including to reshape the distribution of inclusions) is of great potential in many engineering applications.

Since the topic of composite is rather wide, in this section, we will only discuss some special functions that vitrimer is able to offer based on its reversible network. 

#### 3.4.1. Formation and Realignment/Healing of Embedded Magnetic Particle Chains

**Figure 19:**
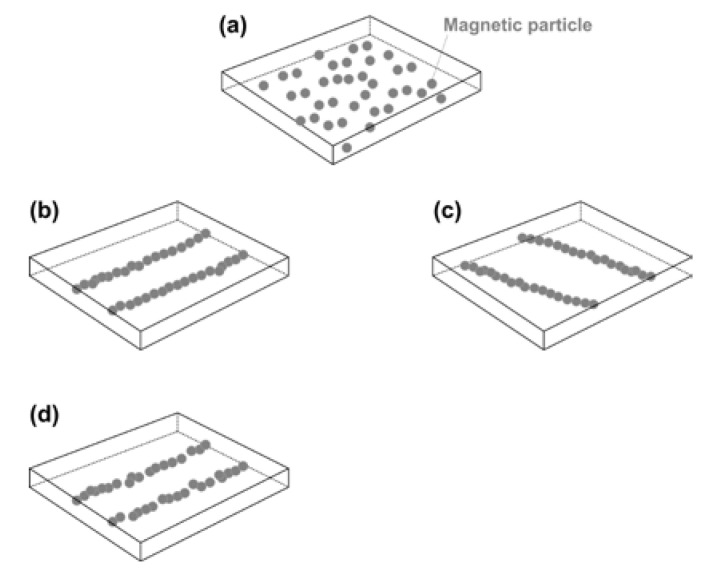
Formation and realignment/healing of magnetic particle chains. (**a**) Randomly distributed magnetic particles; (**b**) chains formed after heating and applying a magnetic field; (**c**) switching of magnetic particle chains upon heating and applying a different magnetic field; (**d**) broken magnetic chains due to, e.g., shape memory cycling.

As illustrated in Figure 19a, we can load magnetic particles (such as, Fe_3_O_4_ powder) into vitrimer via melting mixing or with the help of a solvent (e.g., acetone for the vitrimer-like SMP in Section 2). The randomly distributed particles form regular chains, if a magnetic field is applied when the material is softened (with its shape/dimension maintained) at high temperatures (Figure 19b). Of course, if local heating is applied, chains are only formed within the heated area, which results in tailorable embedded patterns. If conductive-magnetic particles (e.g., nickel, Ni, nano/micro powder) are used, the resulted composite becomes electrically more conductive along the direction of the particle chains, and thus may be heated via joule heating for shape recovery. On the one hand, these magnetic chains are able to switch their direction, if a different magnetic field is applied on the softened material (while the shape maintains) (Figure 19c). Furthermore, the broken magnetic chains, e.g., after shape memory cycling, can be healed in the same way (Figure 19d). According to Figure 9, the magnetic field can be applied after preheating or pre-local-heating at lower temperatures to form chains, which provide great flexibility in processing. Hence, the magnetic particles can be manipulated for reinforcement along a particular direction globally or locally. Refer to Figure S11 (Part III of Supplementary Materials) for an experimental demonstration of the direction switching of the micro sized Ni powder chains embedded inside this vitrimer-like TPU.

#### 3.4.2. Rapid Permanent/Temporary Reshaping with Reinforcement Layer in the Middle 

**Figure 20:**
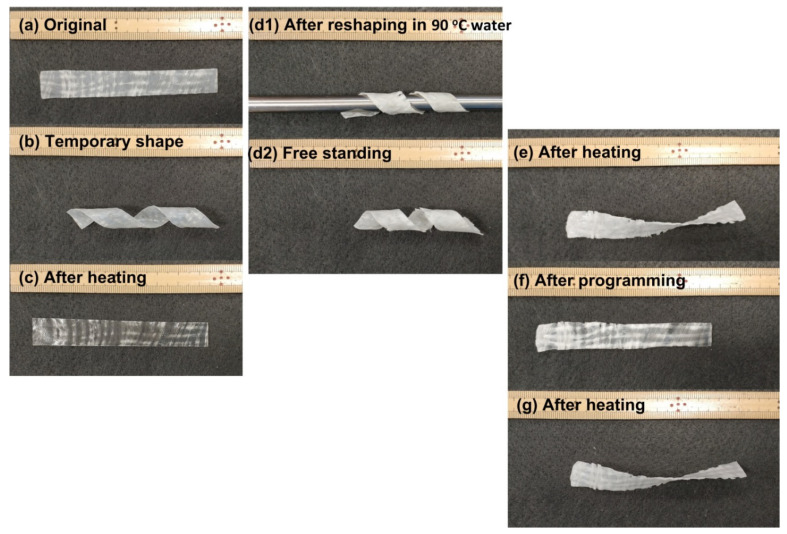
Vitrimer reinforced with glass fabric in the middle (a standard ruler is included as reference). (**a**) Original shape; (**b**) after wrapping around a shaft and heated in about 70 °C water; (**c**) after heating in 80 °C water; (**d1**) after wrapping around a shaft and heated in 90 °C water; (**d2**) free-standing sample after shaft is removed; (**e**) after heating in 80 °C water; (**f**) after programming (flattening) at about 70 °C; (**g**) after heating in 80 °C water for shape recovery.

Glass/carbon fiber-reinforced polymeric composites have been extensively used in many engineering applications [54,55,58,59]. Vitrimer with a fabric layer of glass/carbon fiber in the middle for reinforcement has the advantage of permanent and/or temporary reshaping. Carbon fabric provides the additional convenience in Joule heating of such composites. In Figure 20a, two pieces of the vitrimer-like SMP strips (thickness of each is 0.3 mm) reported in Section 2 are hot-compressed at 110 °C with a layer of commercial glass fabric in between to form a single piece. Subsequently, it is wrapped around a shaft and then heated in hot water of two different temperatures, one is around 70 °C (Figure 20b) and the other is 90 °C (Figure 20(d1)). After cooling back to room temperature, the free-standing shapes of both of them are about the same (Figure 20(b,d2)). After reheating in 80 °C water, one recovers its original flat shape (Figure 20c), while the other appears slightly twisted (Figure 20e). After programming to flatten the twisted piece at about 70 °C (Figure 20f), the sample returns to its twisted shape upon reheating in 80 °C water (Figure 20g), which confirms that this new shape is permanent. According to Figure 7, we can increase the temperature in Figure 20(d1) to achieve better shape fixity. This experiment also demonstrates the feasibility to achieve simultaneous permeant and temporary reshaping based on the cross-linking versus temperature relationship (e.g., Figure 7, Figure 8 and Figure 9 for this particular polymer). 

#### 3.4.3. Comfort Fitting of Wearable Items around Body-Temperature

The heating-responsive shape memory phenomenon in polymers is mostly based on either the glass transition or melting/crystallization. As briefly mentioned above, the temperature ranges for the glass transition of a polymer in the heating and cooling processes are about the same, while the melting temperature range of a polymer is normally much higher than that for crystallization. Thus, we can soften a polymer upon heating to above its melting temperature, and then program it at lower temperatures during the cooling process. If a polymer can be deformed around body temperature or room temperature, while its melting temperature is much higher than the room temperature, this kind of material can be used in comfort fitting around human body temperature. PCL is a good example of such, and has been used extensively in different splints.

Comfort fitting is required in many wearable items [38]. For those items in direct contact with human body, such as splint, the meaning of comfort fitting is two-fold. One is comfort during fitting and the other is comfort after fitting. It is ideal that fitting is carried out at room temperature or around human body temperature. Apart from the requirement on the fitting temperature range, we may need long enough time for fitting as well. PCL is a good material for splints, but lacks flexibility [1]. Hence, after fitting, PCL splints are rigid. 

**Figure 21:**
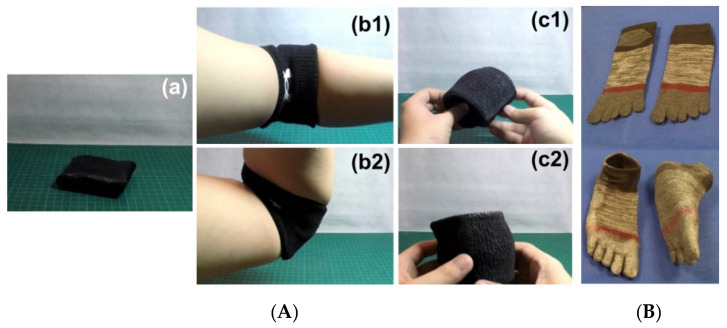
Typical instant comfort fitting items (modified with vitrimer-like SMP). Both were produced by soaking in the acetone solution of this vitrimer-like TPU and then drying in air. (**A**) Flexible elbow band. (**a**) Original (after modification); (**b1**,**b2**) after fitting (with flexibility); (**c1**,**c2**) after being elastically taken off, the programmed shape maintains. (**B**) Toe sock-shoes. Top: original socks; bottom: after modification and fitting (the programmed shape remains after being taken off).

In many wearable items, such as two examples presented in Figure 21, one is elbow band and the other is toe sock-shoes, after modification (either coated with above mentioned vitrimer-like SMP or soaked in its acetone solution and then dried in air), a combination of comfort during and after fitting is achieved. There are more than five minutes for fitting at around body temperature or at room temperature, while the wearable items are still reasonably soft after fitting. Hence, later on they are flexible and can be elastically taken off without much difficulty. The programmed shapes are well maintained even in hot days, unless they are heated to around 60 °C to recover their original shape for next round of fitting. The original shapes of both examples in Figure 21 are actually not permanent, and can always be reshaped upon heating to 110 °C to fix a new permanent shape, since the SMP used here is vitrimer.

**Figure 22:**
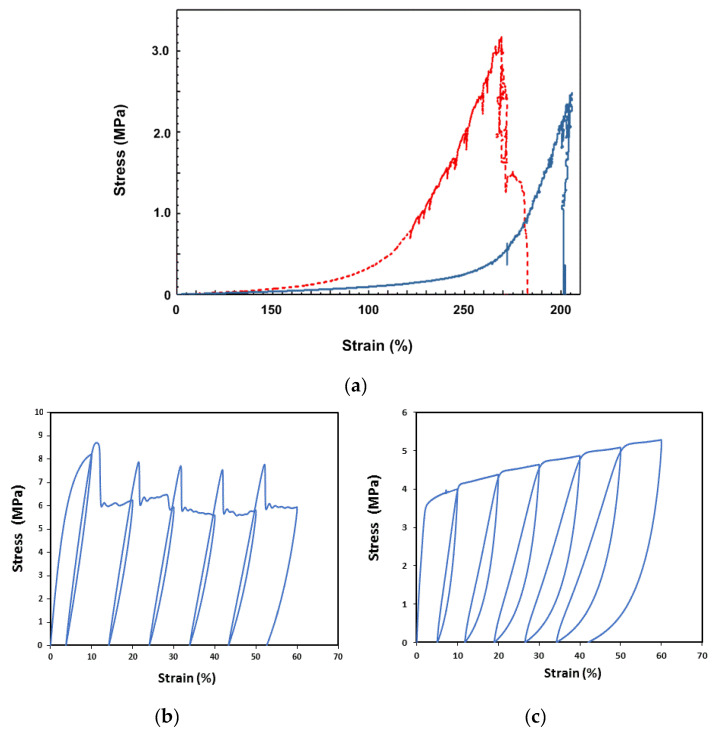
(**a**) Uniaxial stretching of a piece of typical commercial spandex in two in-plane directions. (**b**) Original vitrimer-like SMP (0.3 mm thick) in cyclic stretching to 60% maximum strain with an increment of 10% in each cycle. Apparent necking-propagation phenomenon is observed. (**c**) Cyclic stretching of vtrimer-like SMP (0.3 mm thick) with spandex [as in (**a**)] hot-compressed on both sides. Strain rate: 10^-2^/s (in all tests reported here).

Nowadays, elastic textiles (e.g., spandex) that are highly stretchable in one or two in-plane directions are widely used in sports clothes. In Figure 22a, the stress versus strain relationship of a typical commercial spandex in uniaxial stretching along both in-plane directions is plotted. We can use such a kind of elastic textile to cover one or both sides of a piece of vitrimer-like SMP and then hot-compress into one piece. If the applied temperature is so high that the network is fully eliminated, vitrimer is able to penetrate into the small gaps within textile, which results in strong bonding between textile and vitrimer. The stress versus strain relationships of above investigated vitrimer-like SMP and its composite (0.3 mm thick with both surfaces covered by spandex and hot compressed at 110 °C) under cyclic stretching at room temperature are presented in Figure 22b,c, respectively. The total thickness of the composite is measured and used in the calculation of the stress in Figure 22c. According to Figure 22a, unless an extremely high pressure is applied during hot compression, which causes significant stretching in the elastic textile, the contribution of the elastic textile on the mechanical strength of the resulted composite is practically negligible. Hence, it is not a surprise that the strength of the composite appears to be reduced. In addition, the elastic textile not only smooths the stress versus strain curve (i.e., high yielding peaks are removed), but also changes apparent propagation front movement in the stress plateau range into gradual strain hardening.

**Figure 23:**
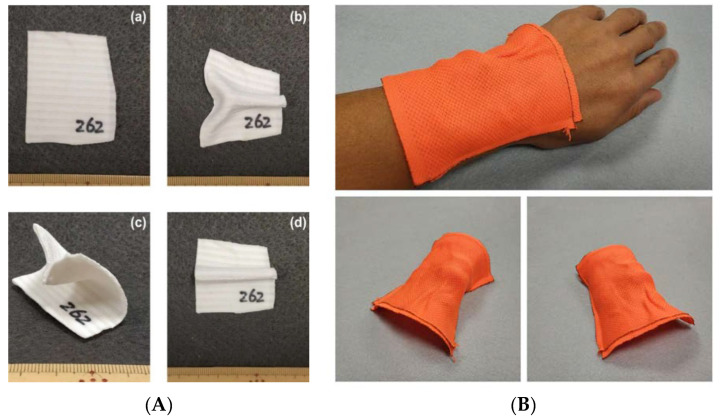
Flexible composites covered with dual-directionally stretchable elastic textile (spandex, in two different colors in I and II, respectively) on both sides. (**A**) Rapid reshaping into another new permanent shape. (**a**) As-fabricated flat piece; (**b**–**d**) different new permanent shapes. (**B**) Fitting at body temperature (temporary shape). Top: on wrist for fitting; bottom: two different angles of view of the programmed free-standing piece after fitting.

This kind of composite can be either reshaped into another permanent shape after heating to 110 °C (Figure 23A) or programmed for comfort fitting at body temperature or below after preheating to less than 80 °C (Figure 23B). Even the associated strain is high in the process of reshaping, the elastic textile (spandex) is soft enough to ensure that its influence is always minimum. On the other hand, after reshaping, either permanent or temporary, for thin plastic composites as mentioned here, they have good flexibility and elasticity for comfort wearing after fitting and can be easily removed in a quasi-elastic manner.

#### 3.4.4. Controlled Unfolding to Minimize Impact

Curved elastic tapes (similar to metallic measuring tape) have been used as elastic hinges for unfolding of deployable structures, such as, solar array panels, in space applications. However, huge impact is produced during unfolding, in particular at the end of the deployment, which results in continuous vibration and/or turbulence of the whole structure that requires additional power and time for stabilization and re-positioning. 

**Figure 24:**
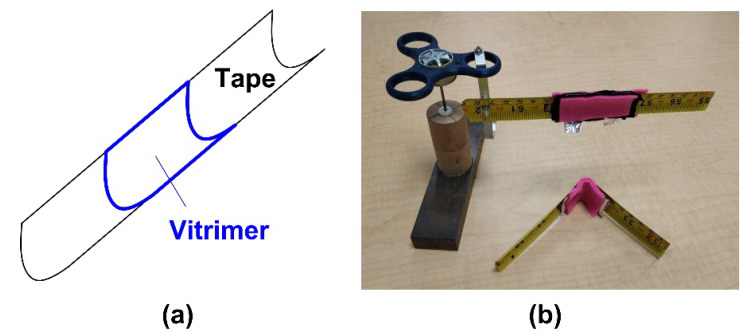
Controlled unfolding upon heating to minimize impact during deployment of elastic hinge. (**a**) Illustration of hinge with vitrimer layer only; (**b**) actual hinges (pink color fabric is used to cover both sides of the bent area for thermal insulation).

A very interesting feature of this vitrimer-like SMP is that after being heated to over 80 °C, it has apparent shear-thickening effect [60], i.e., with the increase in shear rate, its viscosity dramatically increases as well. This effect, which is far more effective than normal damping when a polymer is in the viscous state, helps to minimize the impact in the final deployment stage. As schematically illustrated in Figure 24a, the middle part of a piece of curved elastic tape (represented by a piece of large sized metallic measuring tape) is covered by a vitrimer-like SMP layer (0.3 mm thick) on its inner side [61]. After heating to soften the vitrimer layer, the tape is bent by 90°. Subsequent cooling results in hardening of the vitrimer layer, which is designed to be thick enough to have enough stiffness after cooling/hardening to prevent the elastic tape from recovering (Figure 24b, bottom piece). Upon heating (e.g., via Joule heating) of the vitrimer layer, its restraint on the elastic tape is gradually removed. Hence, recovery (deployment) occurs, but the unfolding speed is slow because of the shear-thickening effect of this vitrimer layer at high temperatures. Thick elastic fabric (in pink color in Figure 24b) is used to cover the bent area for good thermal insulation to save energy in the case of Joule heating for activation. 

### 3.5. Additional Permanent Cross-Linking

#### 3.5.1. Reconfigurable Two-Way Actuation

Two-way (or called reversible) actuation has been achieved in some polymeric materials based on melting/crystallization transition [62,63,64,65]. These materials are able to switch between two shapes, one corresponding to the high temperature shape and the other to the low temperature shape. Same as shape memory alloy-based actuators [66], an elastic stress field, either inside of the polymer or outside (in the form of elastic spring, which also includes elastic structures and constant/variable load), is required for automatic re-programming during thermal cycling. The former (with internal elastic stress field) is called material two-way actuation, and the latter is called mechanical two-way actuation [67].

According to Figure 9, we may heat a pre-deformed sample to partially remove the network, and then cool it to form new network. Consequently, there are two networks in the material, one is the remaining of the previous network, which is pre-strained, and the other is newly formed, which is strain free. Hence, an internal elastic stress field is introduced into the material. Same as the material two-way SME in shape memory alloys [67], such a kind of internal stress field is required for two-way (reversible) actuation of polymers upon thermal cycling without applying any external loading [68]. However, practically it might be difficult to precise control the actual fraction of the new network, since there are many processing parameters involved. 

So far, the most applicable approach to realize material two-way actuation in polymers is to introduce two networks into a semi-crystalline polymer in two steps during curing [69]. The second network is introduced into the polymer after the material is deformed, so that the first network is pre-strained, while the 2^nd^ network is strain free. The resulted polymer is able to switch between two shapes upon thermal cycling. But both high temperature and low temperature shapes are permanent.

Without modifying the chain structure, this vitrimer-like SMP investigated in Section 2 can be cross-linked with dicumyl peroxide (DCP) to form interpenetrating polymer network (IPN) [70]. The resulted polymer is thermoset and has excellent heating-responsive SME even being programmed at 100 °C. Since now there are two networks inside of the polymer, one is permanent (IPN) and the other is reversible (vitrimer), we can reprogram the network associated with the vitrimer-like behavior. Hence, the two-way actuation of the resulted polymer is re-configurable. 

#### 3.5.2. Rapid Additive Manufacturing in Solid State

Current technologies for additive manufacturing (also known as 3D printing) are mostly developed for on-earth environment, which not only relies on the gravitational force, but also requires minimum vibration/disturbance. However, in many situations, e.g., on space missions where gravity is close to zero [71,72], while on air/sea missions (on-board of airplanes/ships) where severe random vibration is unavoidable, it is very hard to use liquid/powder form of raw materials in 3D printing. Even for volumetric additive manufacturing via tomographic reconstruction [73], which is one of the recently developed approaches for rapid 3D printing, good accuracy in cross-linking of polymeric liquid is a challenge, if the printing platform is unstable (such as, on air/sea missions) that normal vibration isolation tables cannot handle. Vitrimer-like SMPs appear to be the right material to realize rapid 3D printing in solid state for above mentioned application scenarios [74], i.e., instead of cross-linking liquid polymers as in current volumetric additive manufacturing via tomographic reconstruction [73], vitrimer-like SMPs can be cross-linked at high temperatures, while they are still in the solid state and transparent. Cross-linking might be UV activated or laser-induced heat activated on space missions or air/sea missions. After cross-linking, the printed model is thermoset, while the uncross-linked part is still vitrimer-like, which can be removed upon heating to the easy to flow temperature (e.g., 120 °C for this vitrimer TPU) or washed away by a special solvent (e.g., acetone for this vitrimer TPU). Any deformation in the model induced during the process to remove the uncross-linked part can be eliminated via activating the SME. Refer to Figure S12 in Part III of Supplementary Materials for a schematic illustration of the major steps in 3D printing.

## 4. Conclusions

Vitrimer-like shape memory polymers (SMPs) combine the vitrimer-like behavior (associated with dissociative covalent adaptable networks) and shape memory phenomenon. This kind of polymers can be utilized to achieve many novel functions that are difficult to be realized by conventional polymers. 

In this paper, we used a commercial polymer to demonstrate how to characterize the vitrimer-like behavior based on the heating-responsive SME. The relationship between the shape memory performance, which is associated with the reversible cross-linking, and pre-process (in particular, thermal history) was obtained for this polymer. Such a kind of information provides the foundation for a series of examples presented here to reveal the potential applications of vitrimer-like SMPs and their composites. 

It can be concluded that apart from conventional applications, such as, re-processability and heat-assisted self-healing, the vitrimer-like feature not only enables many new ways in reshaping polymers (inside/outside, temporarily/permanently, at macroscopic/microscopic scale), but also can bring forward new approaches in manufacturing, such as, rapid 3D printing in solid state.

## Supplementary Materials

The following are available online at https://www.mdpi.com/2073-4360/12/10/2330/s1, Figure S1: Shape memory effect in thermoset polycaprolactone (PCL) [cross-linked with 10 wt. % of benzoyl peroxide (BPO)], Figure S2: Internal stress fields in commercial thermo-plastic PS petri dish (a) and PP box (b), Figure S3: Photo-elasticity images of tough hydrogel (thermoset), Figure S4: Differential scanning calorimetry (DSC) result between −100 °C and 200 °C at temperature ramping rate of 10 °C/min using Netzsch DSC 214 machine (NETZSCH Group, Germany), Figure S5: ^1^H NMR spectrum in acetone-*d6*, Figure S6: XRD spectra recorded at 25 °C, 80 °C and 110 °C upon heating, Figure S7: FTIR spectrum recorded at room temperature, Figure S8: FTIR spectra upon heating from 30 °C to 120 °C (a) in the N-H stretching region and (b) in the C=O stretching region, Figure S9: Schematic illustration of the phase transition process upon heating, Figure S10: Schematic comparison of surface wrinkles formed by surface etching of thermo-plastic (c) and surface preheating of vitrimer (d), Figure S11: Switching of Ni micro powder chains inside vitrimer thin film (about 0.1 mm, produced from acetone solution of this vitrimer-like PU mixed with Ni powder), Figure S12: Rapid 3D printing in solid state on space/sea missions.
