# Supplementary material for: Vitrimer-Like Shape Memory Polymers: Characterization and Applications in Reshaping and Manufacturing
Source: Polymers (Basel). 2020 Oct 12;12(10):2330. doi: 10.3390/polym12102330 (PMC7601385; doi:10.3390/polym12102330)
Supplement: Supplementary file 1 [file polymers-12-02330-s001.pdf]

Supplement

VITRIMER-LIKE SHAPE MEMORY POLYMERS: CHARACTERIZATION AND APPLICATIONS IN RESHAPING AND MANUFACTURING

Tao Xi Wang[a], Hong Mei Chen[b,*], Abhijit Vijay Salvekar[c,$], Junyi Lim[c], Yahui Chen[d], Rui Xiao[e], Wei Min Huang[c,*]

a College of Aerospace Engineering, Nanjing University of Aeronautics and Astronautics, 29 Yudao Street, Nanjing 210016, People's Republic of China; wa0003xi@nuaa.edu.cn (T.X.W.)

b College of Chemistry and Materials Science, Sichuan Normal University, Chengdu 610066, People's Republic of China; chenhongmei@sicnu.edu.cn

c School of Mechanical and Aerospace Engineering, Nanyang Technological University, 50 Nanyang Avenue, Singapore 639798, Republic of Singapore; ABHIJITV001@e.ntu.edu.sg (A.V.S.); li0020yi@e.ntu.edu.sg (J.L.); mwmhuang@ntu.edu.sg (W.M.H.)

d School of Physical Science and Technology, Soochow University, Suzhou 215006, P. R. China; chenyahui@suda.edu.cn

e Key Laboratory of Soft Machines and Smart Devices of Zhejiang Province, Department of Engineering Mechanics, Zhejiang University, Hangzhou 310027, People's Republic of China; rxiao@zju.edu.cn

* Corresponding author. $Email: chenhongmei@sicnu.edu.cn; #Email: mwmhuang@ntu.edu.sg.

$ Currently with Polymerize Pte. Ltd., Republic of Singapore.

Part I

- **Shape memory effect in thermoset PCL**

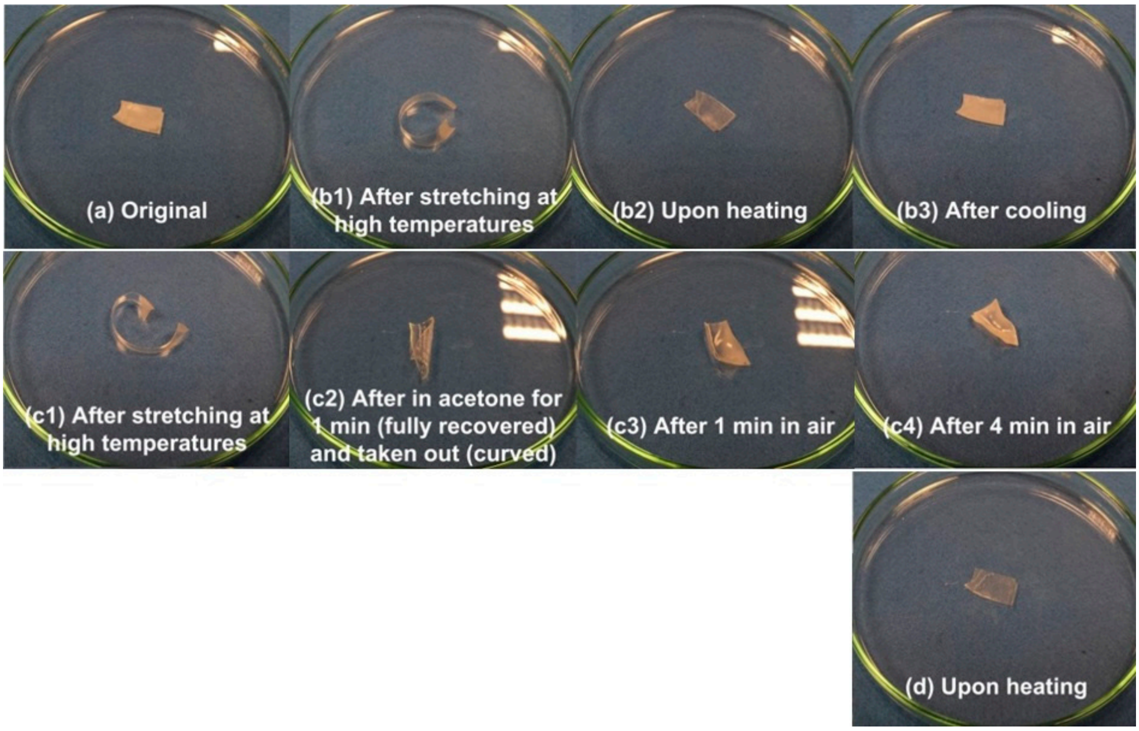

Figure S1 Shape memory effect in thermoset polycaprolactone (PCL) [cross-linked with 10 wt. % of benzoyl peroxide (BPO)]. (a) Original strip shaped sample; (b1)-(b3): heating-responsive SME (after programming via stretching above its melting temperature); (c1-c4): chemo-responsive SME [after programming via stretching above its melting temperature (c1), and then immersing in acetone (c2); (c3-c4) drying in air causing the sample curling up due to rapid and uneven evaporation of acetone]; and (d) after further heating [of (c4)] in hot water to release the internal stress and subsequently to recover its original shape.

- **Internal stress in thermo-plastic and thermoset**

    - **Thermo-plastic (without cross-linking)**

The internal stress in two commercial thermo-plastic items, namely polystyrene (PS) petri dish and polypropylene (PP) box, is examined by conventional photo-elasticity test [1] in Figure S2 (including direct observation via naked eyes of the petri dish). The colourful image of the photo-elasticity test reveals the internal stress field produced by injection moulding. For both pieces, we used a lighter for local heating at one single point, which results in an elliptical shaped small area, indicating the change in the internal stress field. A closer-look reveals that the surface of the locally heated area becomes uneven (e.g., in Figure S2a2) due to overheating induced melting.

- **Thermoset (cross-linked)**

Figure S3(a) is a piece of dried tough hydrogel, which is thermoset and with excellent SME [2]. Although the as-fabricated wet piece is flat [3], it becomes curly as a result of the internal stress that is built up during drying in air [4], which is similar to drying acetone wetted cross-linked PCL in Figure S1(c2-c4). We can flatten it after heating to soften it, since dry hydrogel is essentially a polymer and with the heating/water-responsive SME [5]. The photo-elasticity image of the flattened piece becomes more colourful (Figure S3b). A droplet of water placed on the surface of the sample is able to release the internal stress underneath it, so that after drying, a circular shaped less colourful area is observed (Figure S3c is fully dried after 1st droplet is applied and Figure S3d is fully dried after the 2nd droplet is applied). As a thermoset, its internal stress field can be altered, while the cross-linking prevents the polymer from flowing.

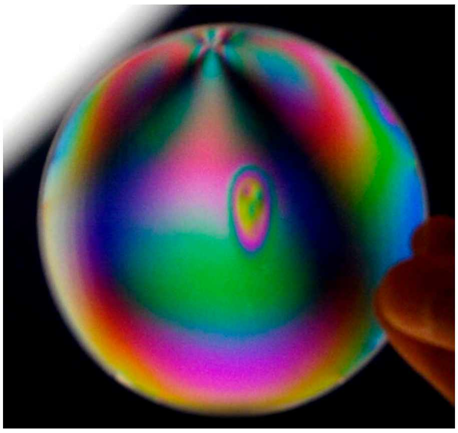

(a1)

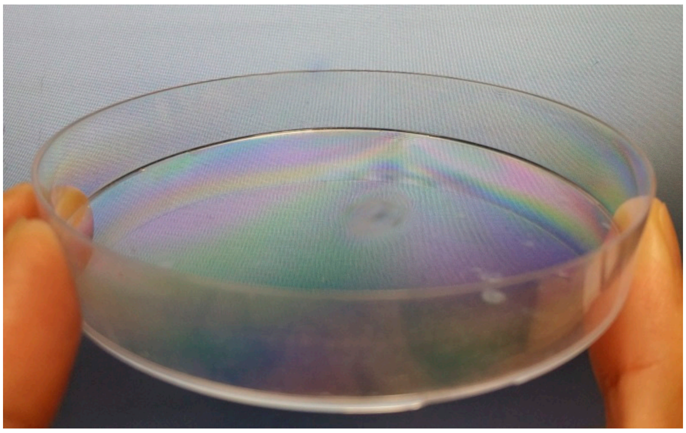

(a2)

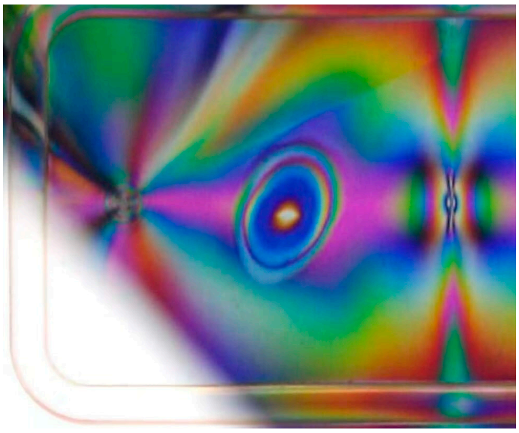

(b)

Figure S2 Internal stress fields in commercial thermo-plastic PS petri dish (a) and PP box (b). (a1) and (b) are images of photo-elasticity (both were placed between a computer monitor and a polarizer covered digital camera); and (a2) [same piece as in (a1)] is an image without polarizer.

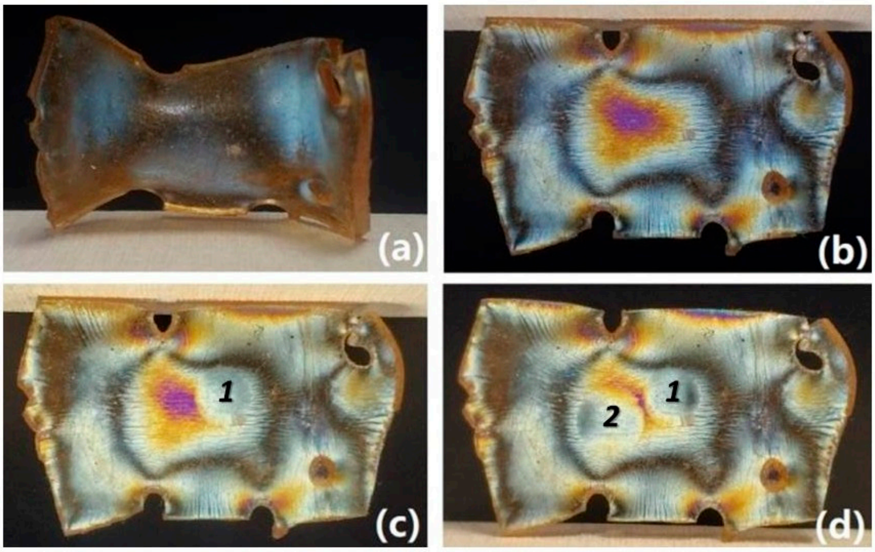

Figure S3 Photo-elasticity images of tough hydrogel (thermoset). (a) Uneven surface after drying in air; (b) after heating and flattening; (c) fully dried in air after 1st water droplet is placed at the central area (marked as 1); (d) fully dried in air after 2nd water droplet is placed at the central area (next to the previous one, marked as 2).

- **Differential scanning calorimetry (DSC) result between -100 ºC and 200 ºC**

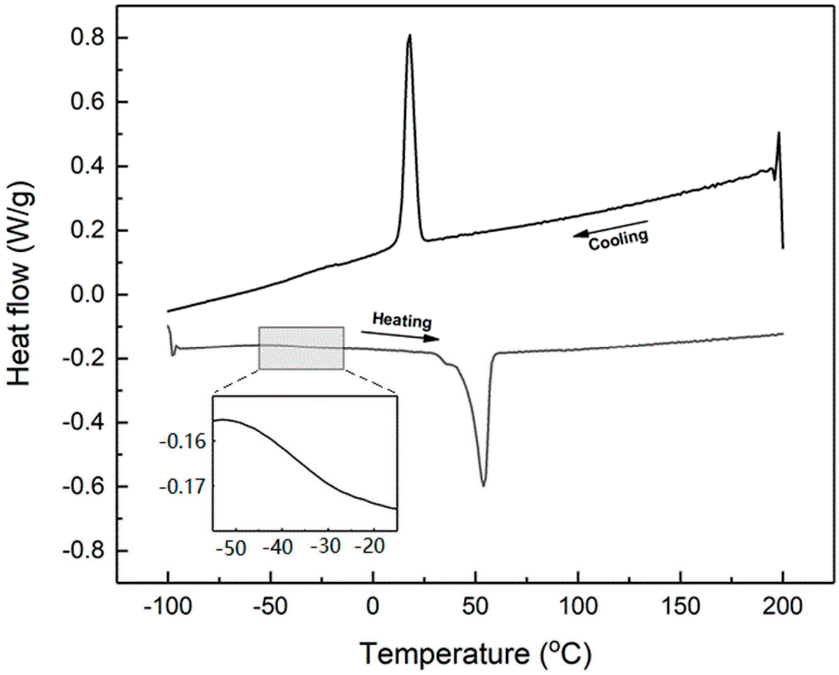

Figure S4 Differential scanning calorimetry (DSC) result between -100 ºC and 200 ºC at temperature ramping rate of 10 ºC/min using Netzsch DSC 214 machine (NETZSCH Group, Germany). Zoom-in view reveals the glass transition is between -50 ºC and -30 ºC.

Part II

A series of additional experiments were conducted in order to identify the chemical structure of this material and subsequently to find the underlying mechanism for the observed vitrimer-like behaviour.

- **Structural characterization**

The structure of this material was analysed by nuclear magnetic resonance (NMR**,** Bruker Unity-400). Tetramethylsilane (TMS) was used as the internal standard and acetone-d_6 was used as the solvent.

According to the ^{1}H NMR spectrum in Figure S5, this TPU is composed of poly(1,4-butylene adipate glycol) (PBA), diphenyl-methane-diisocyanate (MDI), and 1,4-butylene glycol (BDO). PBA segments form the soft domain, while MDI and BDO segments form the hard segments. Based on the areas underneath these peaks, the ratio of soft segments to hard segments (m/n) is determined to be about 28/3.

X-ray diffractometry (XRD) was further used to study the aggregation structure. DSC test was carried out by Rigaku Smartlab with a temperature variable sample cell. The scanning range was 10°−40°. The spectra were collected upon heating the material to 25 ºC, 80 ºC and 110 ºC.

As shown in Figure S6, at 25ºC, obvious diffraction is observed at three 2θ values of 21.76°, 22.42° and 24.02°, which attributes to the crystalline PBA segments in the soft domain. Upon heating to 80 ºC, the diffraction corresponding to crystalline PBA disappears, and amorphous diffraction is observed, which agrees with the DSC result in Figure S4, as the material fully melts at 80 ºC. When the material is heated to 110 ºC, the intensity of amorphous diffraction decreases, which

means that the aggregation interaction in the hard segments decreases when the temperature is increased from 80 ºC to 110 ºC. As determined by the result of the above ^{1}H NMR test, the hard segments are formed by MDI and BOD. We may conclude that the hydrogen bonding interaction formed by N-H and C=O in urethane affects the aggregation interaction in the hard domain.

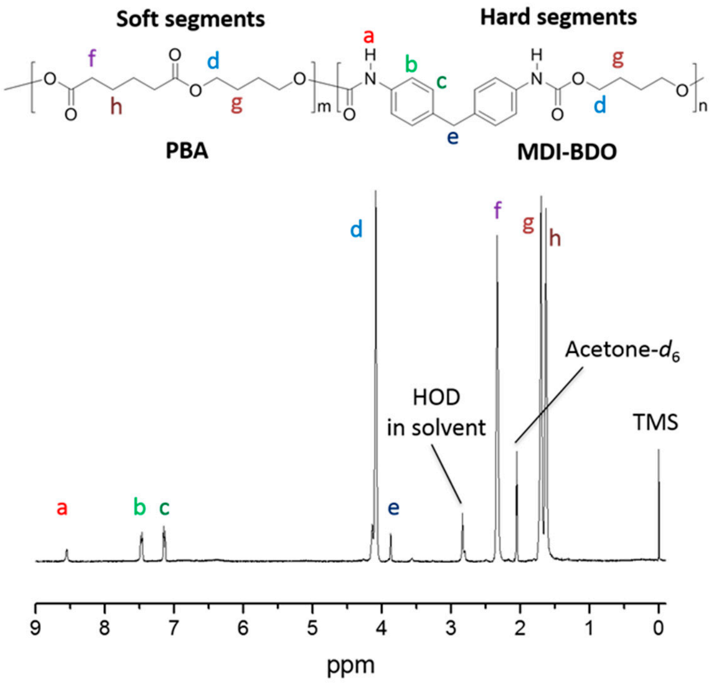

Figure S5 ^{1}H NMR spectrum in acetone-d_6.

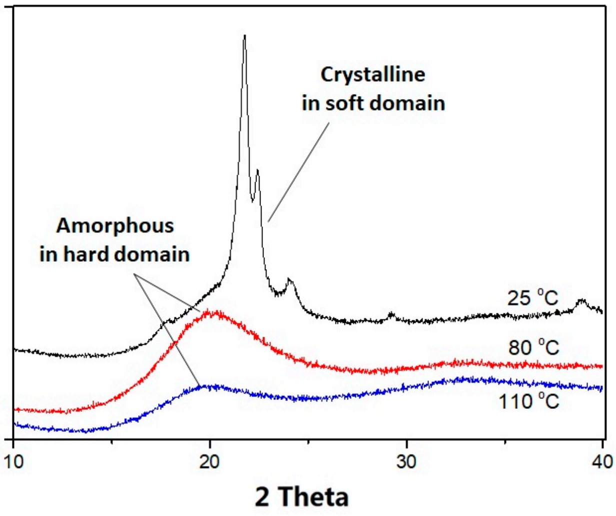

Figure S6 XRD spectra recorded at 25ºC, 80ºC and 110ºC upon heating.

- **Hydrogen bonding interaction**

The hydrogen bonding interaction was further verified using a Fourier-transform infrared spectroscopy (FTIR). The data was recorded by a Bruker Vertex 70 spectrometer at different temperatures (controlled by a Harrick ATC-02402 temperature controller). Dried particles of the material were dissolved in acetone at a concentration of 2% (w/v), and a thin film was prepared by casting the solution on a KBr plate at 60 ºC. Subsequently, this film was held in between two KBr plates in the heating cell and was gradually heated from 30 ºC to 180 ºC. Upon reaching each desired temperature and maintained for one minute, the film was scanned for sixty times at a resolution of 4 cm^{-1}.

Figure S**7** presents the FTIR spectrum of the film recorded at room temperature. This spectrum is mainly characterized by the N-H stretching vibrations at 3500 cm^{-1} - 3200 cm^{-1}, the C-H stretching vibrations at 3100 cm^{-1} - 2800 cm^{-1}, the C=O stretching at 1820 cm^{-1} - 1640 cm^{-1}, the N-H in-plane

bending at 1540 cm^{-1}, and the skeletal vibrations at 1450 cm^{-1} - 1000cm^{-1} and 970 cm^{-1} - 870 cm^{-1}.

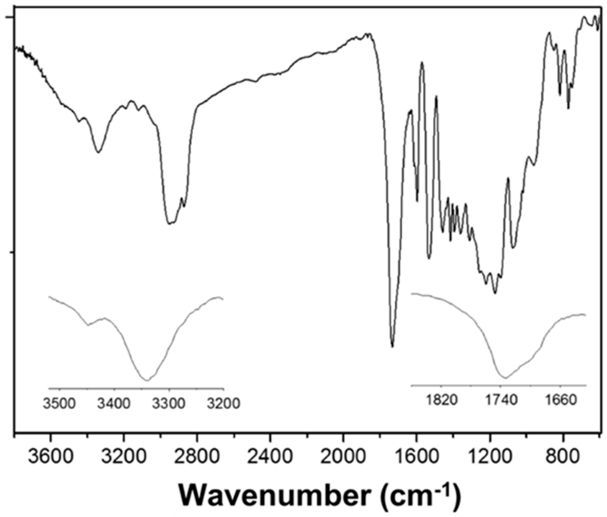

Figure S7 FTIR spectrum recorded at room temperature. Insets are the N-H stretching (3500 cm^{-1} - 3200 cm^{-1}) and the C=O stretching (1860 cm^{-1} -1620 cm^{-1}) regions.

As the focus of this study is on the hydrogen bonding interactions in the hard domain, two spectral regions are of our interest: the N-H stretching vibrations at 3500 cm^{-1} -3200 cm^{-1}, and the C=O stretching vibrations at 1860 cm^{-1} -1620 cm^{-1}.

- **N-H stretching region**

The N-H stretching region in the FTIR spectra presented in the transmittance scale and recorded upon heating is shown in Figure S**8**(a).

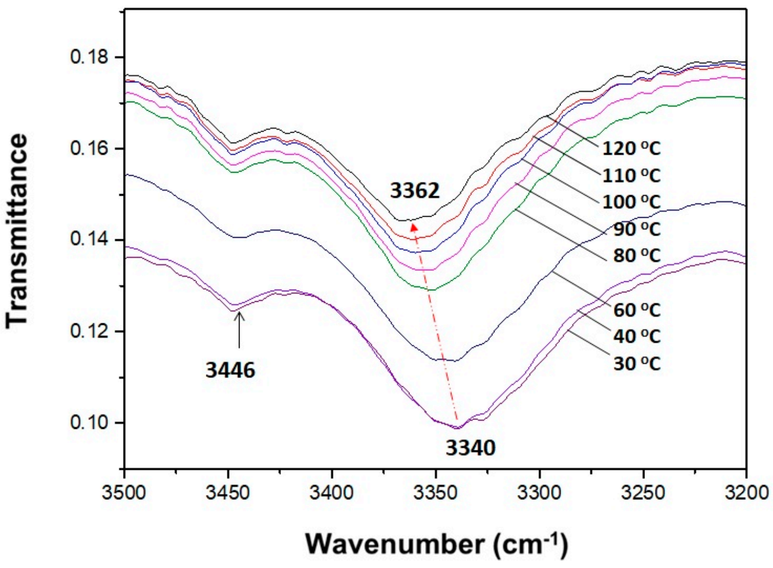

(a)

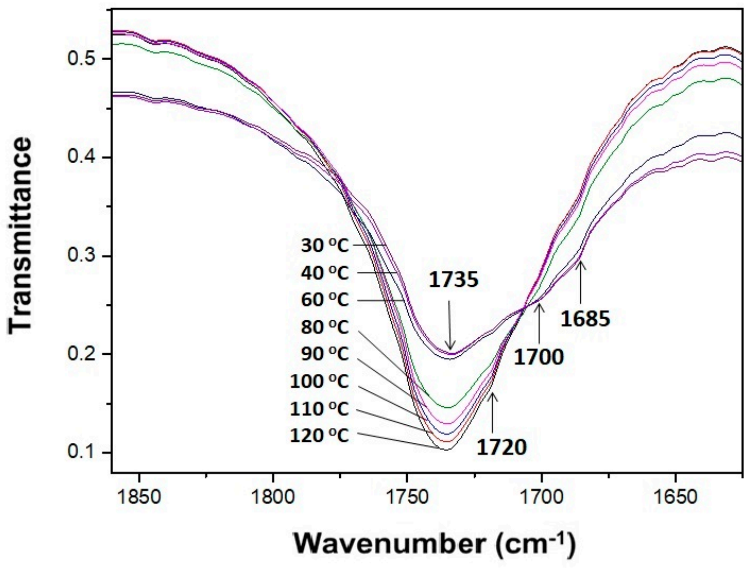

(b)

Figure S8 FTIR spectra upon heating from 30 ºC to 120 ºC (a) in the N-H stretching region and (b) in the C=O stretching region.

The infrared bands at 3446 cm^{-1} and 3340 cm^{-1} are assigned to be the stretching modes of the “free” and hydrogen bonded N-H groups, respectively, at 30 ºC. Upon heating from 30 ºC to 120 ºC,

while the peak position for the "free" N-H stretching band maintains almost at the same position of 3446 cm^{-1}, the intensity for the "free" band keeps the same as the temperature is increased from 30 ºC to 120 ºC. This is similar to what is reported in [6]. The peak position and intensity of the hydrogen bonded N-H stretching upon heating demonstrate a little different trend. The peak position remains constant from 30 ºC to 60 ºC, and then shifts from 3340 cm^{-1} to 3362 cm^{-1} in the temperature range from 80 ºC to 120 ºC, which indicates the decrease in the strength of the hydrogen bonds as the temperature is above 80 ºC. The intensity of hydrogen bonded N-H stretching stays high and almost constant at below 60 ºC, and a steep decrease takes place in the temperature range from 80 ºC to 120 ºC. The sharp decrease indicates that the hard domains gradually become amorphous.

- **C=O stretching region**

According to above structural analysis, the C=O stretching bands should include two components, namely, the ester C= O stretching and the urethane C=O stretching. The latter is further composed of three subcomponents corresponding to the "free", disordered hydrogen bonded, and ordered hydrogen bonded C=O stretching, respectively. The "free" C=O groups are the ones forming non-hydrogen bonds, whereas the disordered and ordered hydrogen bonded ones are the hydrogen bonded groups associated with the amorphous phase and crystalline phase, respectively.

Figure S**8**(b) reveals the FTIR spectrum in the C=O stretching region in the transmittance scale upon heating. Only a wide band is observed at 1820 cm^{-1} to 1640 cm^{-1}, which may attribute to the overlapping of the ester C=O stretching in the range of 1780 cm^{-1} to 1740 cm^{-1} and the urethane C=O stretching in the range of 1740 cm^{-1} to 1660 cm^{-1}. Upon heating, the shift of peak position and the change in the shape of the peak are apparent. The band at 1720 cm^{-1} is attributed to the

“free” C=O groups, the band at 1700 cm^{-1} is the disordered hydrogen bonded C=O, and the band at 1685 cm^{-1} is the ordered hydrogen bonded C=O. The bands at 1700 cm^{-1} and 1685 cm^{-1} can be easily identified upon heating to 60 ºC, and then both bands gradually disappear upon further heating, which means that the intensity of hydrogen bonded C=O decreases.

- **Underlying mechanism for vitrimer-like behavior**

At this point, we may conclude that the hydrogen bonding interactions in the hard domain are kept constant upon heating to 60 ºC. Upon further heating to above 80 ºC, the interactions gradually decrease.

Now, the phase transition process upon heating, which is associated with the observed vitrimer-like behaviour in this material, may be schematically sketched as shown in Figure S9. At room temperature (around 25 ºC), the soft segment of this material is crystalline, while the hard segment is glassy. Upon heating to around 60 ºC, the soft segment melts, and the hard segment remains glassy. Hence, the material is similar to a semi-crystalline polymer [7], and the hard segment serves as the elastic part, while the soft segment functions as the transition part for the heating-responsive SME [8]. Upon further heating to over 80 ºC, the hard segment gradually becomes viscous, so that the shape recovery ratio gradually decreases. At around 120 ºC, the material turns out to be thermo-plastic, since now it is able to flow even under the gravitational force. Upon cooling, the hard segment gradually becomes glassy, and the soft segment starts to crystallize at lower temperatures.

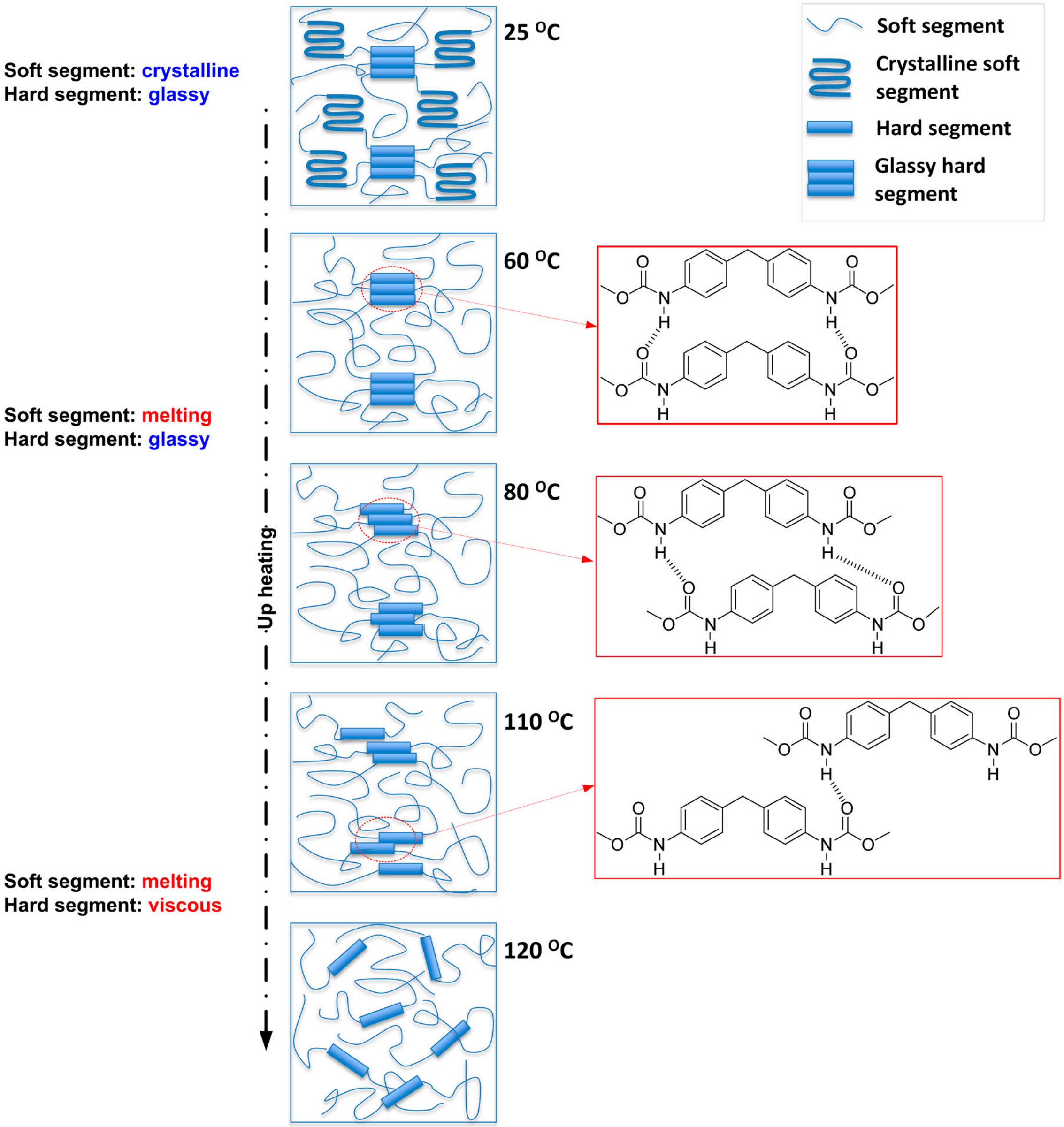

Figure S9 Schematic illustration of the phase transition process upon heating.

In addition, instead of heating, acetone is able to eliminate the bonding between urethane C=O and N-H, resulting in the material to be dissolved in acetone. Upon drying, acetone evaporates, so that the bonding between urethane C=O and N-H is re-formed.

Part III

- **Schematic comparison of surface wrinkles formed by surface etching of thermo-plastic and surface preheating of vitrimer**

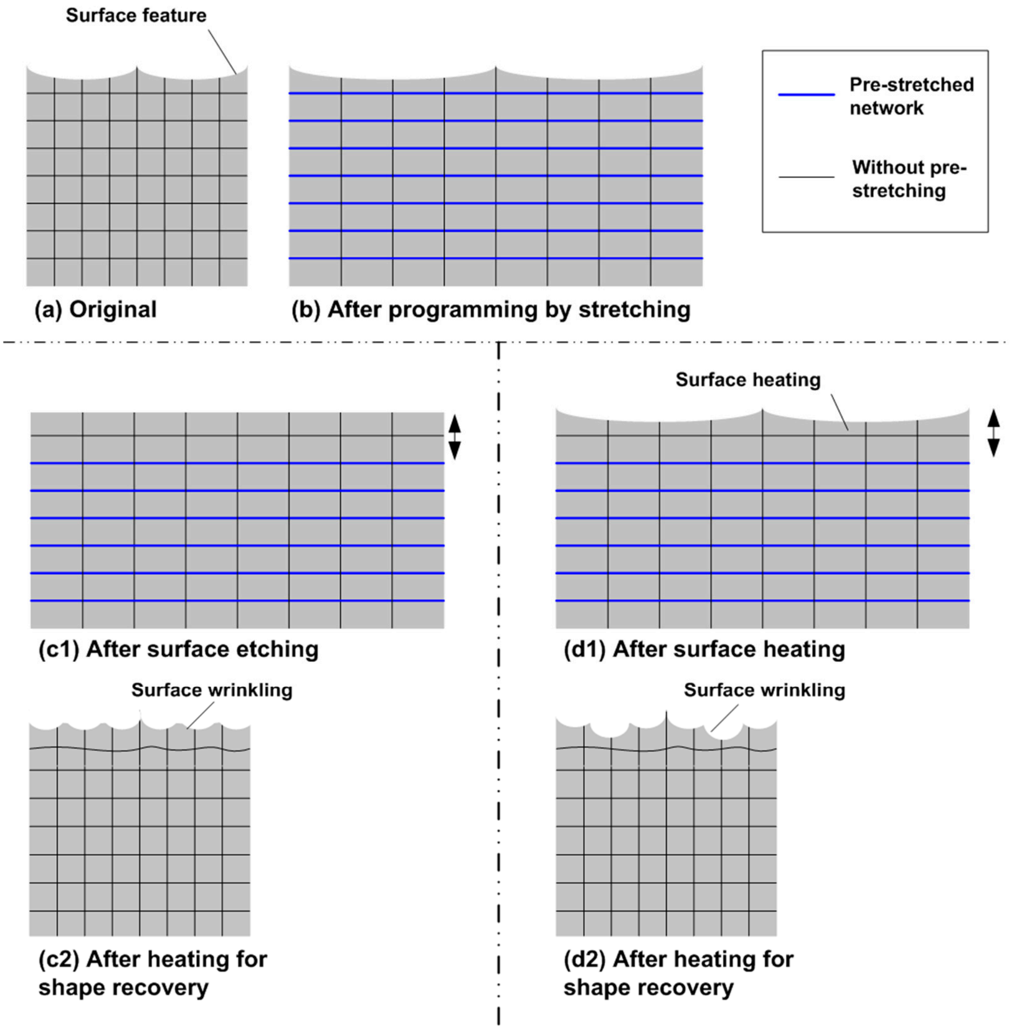

Figure S10 Schematic comparison of surface wrinkles formed by surface etching of thermo-plastic (c) and surface preheating of vitrimer (d). (a) Original (with initial surface pattern); (b) after programming via stretching; (c1) after surface etching; (d1) after surface preheating; (c2 and d2) after heating for shape recovery.

In Figure S10, we schematically compare the difference between surface etching (thermo-plastic) approach and surface preheating (vitrimer) approach. While etching smooths the surface of

thermo-plastic (Figure S10c1), surface preheating of vitrimer, if processed carefully according to the features of a particular material, is able to mostly keep the initial surface feature (pre-deformed) (Figure S10d1). Therefore, the resulted surface feature of the surface etched thermo-plastic is parallel wrinkles only (Figure S10c2), while the final surface feature of the surface preheated vitrimer is a combination of the initial surface pattern (pre-deformed) and newly formed parallel wrinkles (Figure S10d2).

- **Switching of Ni micro powder chains inside vitrimer**

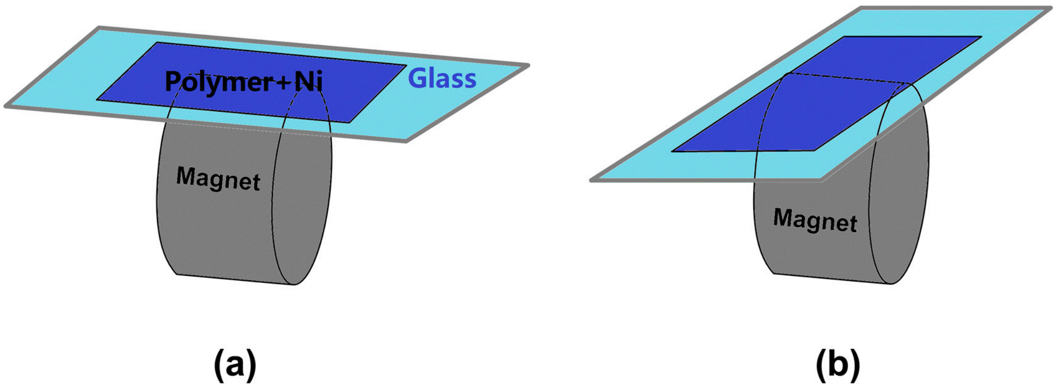

(I)

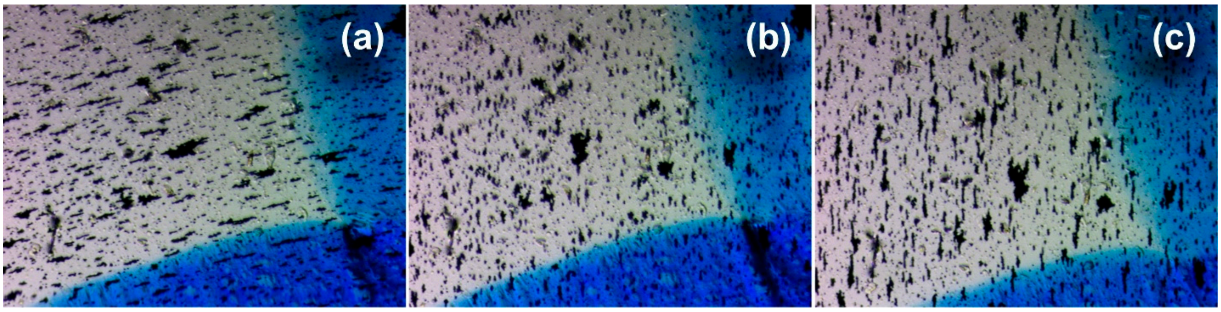

(II)

Figure S11 Switching of Ni micro powder chains inside vitrimer thin film (about 0.1 mm, produced from acetone solution of this vitrimer-like PU mixed with Ni powder). (I) Experimental setup. (a) A thin layer of vitrimer TPU (with Ni micro powder inside) is coated on glass (1 mm thick). It is heated to about 95 °C and then placed atop a cylindrical magnet (7 mm in diameter, 2.5 mm thick) as shown to form Ni chains; (b) heating to about 95 °C again and then placing atop the magnet as shown (turning about 90°). (II) Results (under a microscope, the width of each image is 1 mm, part of the area is marked using a blue permanent pen for easy to spot the same area). (a) After I(a) and cooling for 30 mins; (b) after II(b) and cooling for about 3 mins; (c) after II(b) and cooling for another 30 mins.

- **Rapid volumetric additive manufacturing in solid state**

The main steps are as following.

1. Heating a vitrimer-like SMP into transparent but still solid state (Figure S12a).
2. Cross-linking by UV or laser induced heat on space missions (Figure S12b1) or sea missions (Figure S12b2).
3. After cross-linking, the printed model is thermoset and is within the vitrimer-like SMP (Figure S12c).
4. The uncross-linked part (vitrimer) may be removed upon heating or using a special solvent. Any deformation in the model induced during the process to remove the uncross-linked part can be eliminated via activating the SME (Figure S12d).

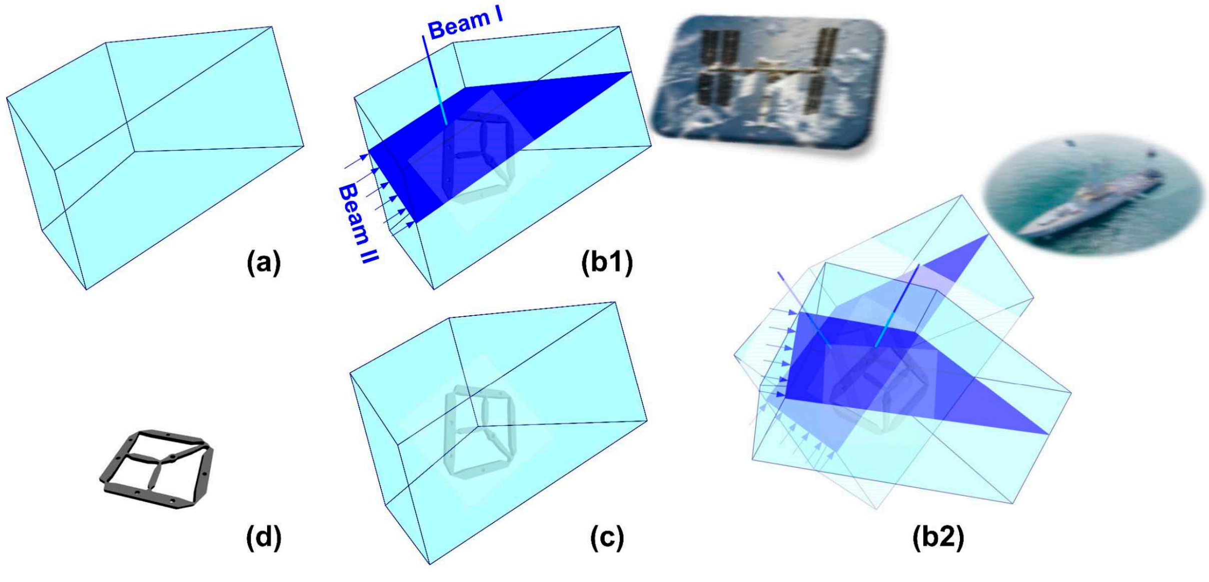

Figure S12 Rapid 3D printing in solid state on space/sea missions. (a) High temperature vitrimer in solid state; (b1) dual-beam cross-linking in space-station (microgravity); (b2) dual-beam cross-linking on battleship (random vibration); (c) after printing; (d) after removal of the uncross-linked part.

References

[1] Bradley W, Kobayashi A. An investigation of propagating cracks by dynamic photoelasticity. Experimental Mechanics. 1970;10:106-13.

[2] Zhang JL, Huang WM, Gao G, Fu J, Zhou Y, Salvekar AV, et al. Shape memory/change effect in a double network nanocomposite tough hydrogel. European Polymer Journal. 2014;58:41-51.

[3] Wang Q, Hou R, Cheng Y, Fu J. Super-tough double-network hydrogels reinforced by covalently compositing with silica-nanoparticles. Soft Matter. 2012;8:6048-56.

[4] Huang WM, Lu HB, Zhao Y, Ding Z, Wang CC, Zhang JL, et al. Instability / collapse of polymeric materials and their structures in stimulus-induced shape / surface morphology switching. Materials and Design. 2014;59:176-92.

[5] Zhang JL, Huang WM, Lu HB, Sun L. Thermo-/chemo-responsive shape memory/change effect in a hydrogel and its composites. Materials & Design. 2014;53:1077-88.

[6] Wang W, Wang W, Chen X, Jing X, Su Z. Hydrogen bonding and crystallization in biodegradable multiblock poly (ester urethane) copolymer. Journal of Polymer Science Part B: Polymer Physics. 2009;47:685-95.

[7] Mather PT, Luo XF, Rousseau IA. Shape memory polymer research. Annual Review of Materials Research. 2009;39:445-71.

[8] Huang WM, Zhao Y, Wang CC, Ding Z, Purnawali H, Tang C, et al. Thermo/chemo-responsive shape memory effect in polymers: a sketch of working mechanisms, fundamentals and optimization. Journal of Polymer Research. 2012;19:9952.
